# DnaK as Antibiotic Target: Hot Spot Residues Analysis for Differential Inhibition of the Bacterial Protein in Comparison with the Human HSP70

**DOI:** 10.1371/journal.pone.0124563

**Published:** 2015-04-23

**Authors:** Federica Chiappori, Marco Fumian, Luciano Milanesi, Ivan Merelli

**Affiliations:** 1 Institute of Biomedical Technologies, National Research Council (CNR), Segrate (Mi), Italy; 2 Department of Biotechnology and Biosciences, University of Milano-Bicocca, Milano, Italy; University of Westminster, UNITED KINGDOM

## Abstract

DnaK, the bacterial homolog of human Hsp70, plays an important role in pathogens survival under stress conditions, like antibiotic therapies. This chaperone sequesters protein aggregates accumulated in bacteria during antibiotic treatment reducing the effect of the cure. Although different classes of DnaK inhibitors have been already designed, they present low specificity. DnaK is highly conserved in prokaryotes (identity 50–70%), which encourages the development of a unique inhibitor for many different bacterial strains. We used the DnaK of *Acinetobacter baumannii* as representative for our analysis, since it is one of the most important opportunistic human pathogens, exhibits a significant drug resistance and it has the ability to survive in hospital environments. The *E*.*coli* DnaK was also included in the analysis as reference structure due to its wide diffusion. Unfortunately, bacterial DnaK and human Hsp70 have an elevated sequence similarity. Therefore, we performed a differential analysis of DnaK and Hsp70 residues to identify hot spots in bacterial proteins that are not present in the human homolog, with the aim of characterizing the key pharmacological features necessary to design selective inhibitors for DnaK. Different conformations of DnaK and Hsp70 bound to known inhibitor-peptides for DnaK, and ineffective for Hsp70, have been analysed by molecular dynamics simulations to identify residues displaying stable and selective interactions with these peptides. Results achieved in this work show that there are some residues that can be used to build selective inhibitors for DnaK, which should be ineffective for the human Hsp70.

## Introduction

Heat Shock Proteins (Hsp) are essential for the survival cells and their expression levels rely on cellular conditions. In particular, proteins belonging to the Hsp70 family are involved, under stress conditions, in signal transduction, cell cycle regulation, and programmed cell death. Other conditions that involve these proteins are principally native protein folding, refolding and prevention of protein aggregation [1]. Their essential role for pathogenic microorganisms growing in a host is of particular interest for drug discovery. DnaK belongs to the Hsp70 family and is the bacterial homolog of human Hsp70. In particular, DnaK displays up to 70% of sequence identity with respect to the other eukaryotic proteins of this family [2].

DnaK has been characterized in several pathogenic bacteria and seems to have important functions in stress resistance and pathogenicity in multiple-drug-resistant bacteria, such as *Acinetobacter baumannii* [3–4], which is one of the most important opportunistic human pathogens displaying several antibiotic resistances. The heat-shock response, and in particular the DnaK machinery, is involved in the antibiotic resistance mechanism of *A baumannii* [5]. In detail, it results necessary for bacteria survival in unfavourable conditions, such as exposure to oxidative stress, nutrient limitation, extreme temperatures, and presence of heavy metals or antibiotics [6–8]. DnaK mutations increase the bacterial sensitivity to fluoroquinolones, oxacillin and methicillin in normally resistant strains [6–8], since this protein sequesters the aggregates that accumulate in cells exposed to these antibiotics [6] and assists the refolding of proteins misfolded after a stress event [9].

**Fig 1:**
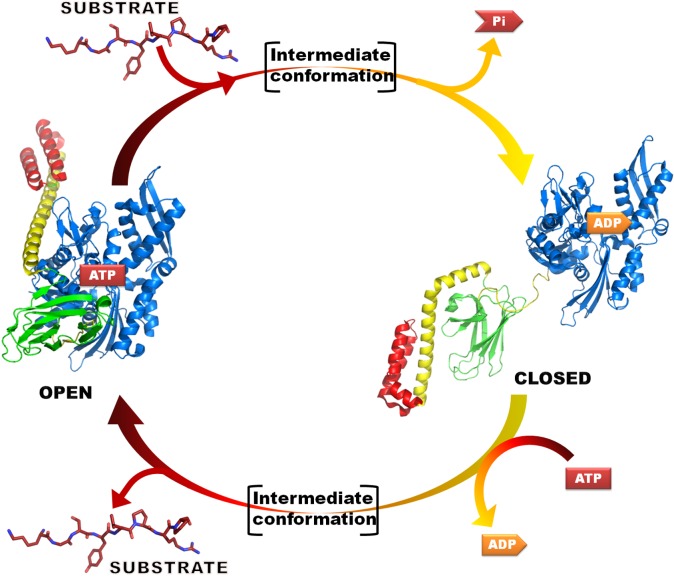
Schematic representation of the Hsp70 allosteric cycle. NBD is in blue, βSBD is in green, helices HA and HB are in yellow and helices HC-HE are in red.

Like all other Hsp70 proteins, DnaK is composed of about 650 residues, arranged in two domains: the nucleotide binding domain (NBD) and the substrate binding domain (SBD), these are connected by a highly flexible linker involved in the allosteric communication between the two domains. When the NBD domain hydrolyses an ATP molecule, the SBD domain assumes a closed conformation, which binds a short extended hydrophobic polypeptide sequence [10]. Therefore, DnaK displays two extreme conformations (Fig 1): in the open state, ATP is bound to the NDB cavity and the substrate affinity is low, while in the closed conformation, after the ATP hydrolysis, the affinity for the substrate is high. Furthermore, the binding of peptides to the SBD induces the ATP hydrolysis in the NDB and the ADP presence induces the SBD rearrangement to the closed conformation, which correspond to a ~10 fold affinity increase for the peptides [11]. The nucleotide exchange from ADP to ATP induces the SBD opening and the substrate release, this brings back the protein to the open conformation (Fig 1). The open/closed state rearrangement depends on the nucleotide that is bound to the NBD domain: in particular, the ATP-bound DnaK is characterized by a low affinity and a fast exchange rate for the substrate, while the ADP-bound form displays a high affinity and low exchange rates. To complete the allosteric cycle, two classes of cochaperone help DnaK/Hsp70 proteins, respectively the DnaJ/Hsp40 and the GrpE/Hip. DnaJ has a J-domain that presents the substrate to DnaK and induces its ATPase activity, resulting essential for the DnaK functionality, while GrpE is a nucleotide exchange factor that increases the basal ADP/ATP exchange rate of DnaK [10].

SDB consists of two sub-domains, βSBD and αSBD. The former is a β-sandwich of two antiparallel β–sheets composed of four strands each one and connected by seven loops. Among these loops, L_1,2_ and L_3,4_ in combination with strands 1 and 2 conform the deep peptide binding cavity. αSBD consists of five helices, one of which, indicated as HB, forms a lid over the polypeptide binding site [12]. The binding cavity is mainly hydrophobic and regions surrounding the cavity are negatively charged [9].

Short hydrophobic peptides (~7 residues) were identified by experimentally screening libraries of compounds able to bind SBD [13]. The hydrophobic core of these peptides is composed of 2–4 residues containing Leu and excluding acid residues, also from the flanking regions [9, 13]. A general core motif ±HyHyHyHyHy± (where ± stands for positively charged residues and Hy refers to hydrophobic residues Leu, Ile, Val, Ala, Gly) was identified [13–15]. Interactions between the peptide-substrate and the SBD binding site involve, respectively, the side-chains of the peptide in van der Waals and hydrophobic contacts, and the peptide backbone in hydrogen bonds with the cavity-forming loops [9, 13]. Once the lid is closed on the substrate, other interactions with the helices (electrostatic and van der Waals) stabilize the conformation [14].

**Fig 2:**
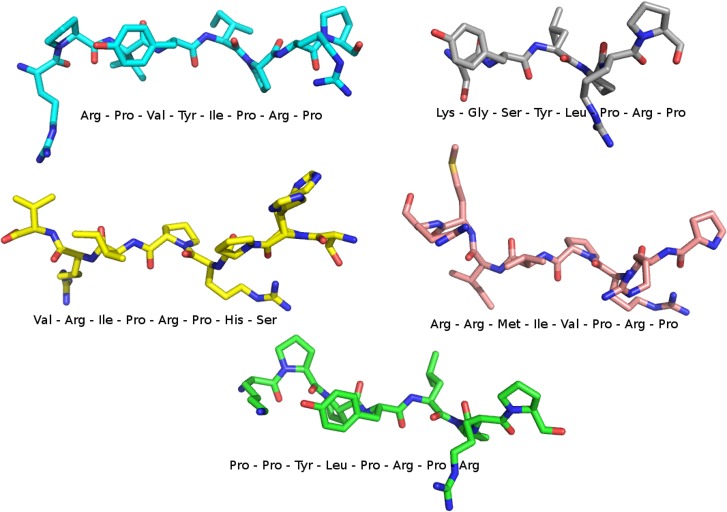
Structures and sequences of the five peptides employed in this study. Atoms are coloured by atom type.

Recently some peptides with antibacterial characteristics have been isolated in insects. The optimized forms of these peptides able to bind DnaK are relatively stable to protease digestion, due to the high proline content, are nontoxic to human cell lines, and are predominantly active against Gram-negative bacteria [2, 16]. In particular, there are few well-known peptides that inhibit DnaK, which are Api88, drosocin, heliocin, oncocin and pyrrhocoricin (Fig 2). These peptides are composed of 18–22 amino acids capable of binding the SBD and keeping the lid in a closed position, preventing the substrate release [8]. Unfortunately, these peptides display low specificity for DnaK: for example, api88 shows a pro-apoptotic activity in DnaK-null cells and it is supposed to bind GroEL [17]. Moreover, peptide drugs generally suffer from their intrinsic metabolic instability [18].

In this study, we identify SBD hot spot residues that can be exploited for the differential inhibition of the bacterial DnaK in comparison to the human Hsp70. The key residues for the interaction with the inhibitor-peptides were recognized, selecting residues conserved in bacteria and not-conserved in the human Hsp70 family. The study is based on sequence analysis, evaluation of the binding site through molecular dynamics (MD) and interaction analysis. MD is a good approximation for studying protein-peptide binding with atomistic detail, and to take into account the dynamic behaviour of the whole system [14, 19]. In detail, three proteins, *A*.*baumannii* DnaK, representative for the opportunistic human pathogens, *E*.*coli* DnaK, included as reference and control structure, and human Hsp70-2 in closed conformation, were simulated in complex with Api88, drosocin, heliocin, oncocin, and pyrrhocoricin bound to the SBD. Results achieved for the bacterial complexes were compared to data obtained for the human model. Our analysis resulted in a set of key residues of the DnaK binding site that are involved in stable interactions with the ligands. Moreover, we developed a pharmacophore model, which can be employed in a further virtual screening study to identify ligands with high specificity and selectivity.

## Materials and Methods

### Sequence analysis

Protein sequences were downloaded from Uniprot [20]. Bacterial sequences were obtained searching for the “DnaK” gene name in the “reference proteome” text box and only reviewed sequences within the taxonomy group “Bacteria” were chosen. Sequences were aligned with Clustal Omega [21] using default settings. Moreover, the SIAS web server [22] was employed to calculate pairwise identities and similarities of multiple sequence alignments, taking gaps into account. WebLogo [23] was employed to obtain a graphical representation of residue frequencies in the βSBD and HA- HB-SBD positions.

### Structure

The closed conformation of *E*.*coli* DnaK in complex with ADP-Mg^2+^ (pdb ID: 2KHO) [24] was obtained from the Protein Data Bank [25]. Structural models of *A*.*baumannii* DnaK and human Hsp70-2 were obtained relying on *E*.*coli* DnaK in closed conformation, using the Swiss-model server [26].

Coordinates of the inhibitors were extracted, respectively, from the X-ray structure of *E*.*coli* SBD bound to Api88 (pdb ID: 4E81) [16], Drosocin (pdb ID: 4EZR) [11], Heliocin (pdb ID: 4EZT) [11], Oncocin (pdb ID: 3QNJ) [5] and pyrrhocoricin (pdb ID: 4EZN) [11]. These peptides were superimposed to the whole structures of *E*.*coli* and *A*.*baumannii* DnaK and human Hsp70 to obtain the complexes to simulate. Peptides are composed of 18–22 amino acids, but for each inhibitor only the 8 core-residues were co-crystallized and therefore included in our complexes.

### Molecular dynamics

MD simulations were performed with GROMACS 4.0 [27], by employing the GROMOS96 (ff43a1) force field [28]. Each complex was included in a rhombic dodecahedric box filled with water (TIP3P model), setting the distance between the complex and the box walls to 1nm. Na^+^ ions were added to complexes for neutralizing the net charge of the system. All complexes were energy relaxed with 1000 steps of “steepest-descendent” energy minimization. MD simulations were performed using the LINCS algorithm [29] to constrain bond lengths, while periodic boundary conditions were applied in all directions. Long-range electrostatic forces were treated using the Fast Particle-Mesh Ewald method (PME) [30]. Van der Waals forces and Coulomb potential were treated using a cut-off distance of 0.9nm and the neighbour list was updated every 5 steps. The simulation time step was set to 2fs and coordinates were saved every 10 ps. An initial velocity was given to all atoms according to a Maxwell distribution at 300K. All simulations were run in an NVT environment employing V-rescale as temperature coupling algorithm, with reference temperature set at 300K.

### Molecular dynamics analysis

Analyses of MD simulations were performed using different software from the GROMACS package. System stability was verified analysing the energy components and the RMSD of the trajectory conformations compared to the starting structure. The cluster analysis was performed using the *g_cluster* module of GROMACS to obtain representative conformations of protein complexes. Clustering of the trajectories was achieved fitting C_α_ atoms with the gromos method [31], with a cut-off distance of 0.5 nm. Trajectories were visualized with VMD [32].

Contact maps were generated using the *g_mdmat* tool of GROMACS, which identifies the minimum distance between residues by calculating the smallest distance between any pair of their atoms. A truncation distance of 0.5 nm was employed to obtain the contact map. This analysis was useful for identifying residues in the neighbourhood of inhibitor-peptides during simulations.

The interaction analysis was performed with the *g_hbond* module of GROMACS, applied to MD simulations, while in-house developed scripts were employed to identify key residues for the interaction with inhibitor-peptides [33]. In particular, both hydrogen bonds and electrostatic contacts were evaluated during the whole trajectories. Starting from the H-bonds existence matrix, the fraction of time that a particular H-bond or contact is present during the trajectory was calculated using the script *plot_hbmap*.*pl* [34]. Residues involved in hydrogen bonds for at least 25% of the trajectory (contacts for at least 50%) were considered for the analysis. Among these residues, only amino acids conserved in the bacterial DnaK and not in the human Hsp70 were filtered. For contacts analysis, cut-offs were set to 0.39 and 0.41, respectively, as set in a previous work [35].

Computational alanine-scanning was accomplished by employing the Robetta server [36–38], which uses Rosetta as backend software. In particular, Rosetta alanine-scanning individually substitutes all residues at the protein-protein (or protein-peptide) interface with alanine and estimates the change in binding free energy.

Structure-based pharmacophore models were generated with LigandScout [39]. Interactions between ligands and proteins were automatically analysed considering both electrostatic and geometric (distances, size, and angles) properties of the binding regions. In particular, hydrophobic anchoring points, H-bond acceptors/donors, positively or negatively ionizable/charged groups, and aromatic rings involved in aromatic interactions were evaluated for building the pharmacophore. As a result, the spatial distribution of the pharmacophoric feature points, which represents the protein residues involved in the interaction, was obtained. We achieved a pharmacophore model for each DnaK-peptide complex representative conformation identified in the cluster analysis of the MD trajectories. Pharmacophore models were aligned and only common characteristics were considered in the final model.

A previously developed approach was applied to estimate the enthalpy variation caused by the ligand binding [35] to the protein, which can also be decomposed in the individual contribution of each residue include in the binding sites. In particular, the Lennard-Jones and Coulomb terms were estimated using the Gromacs tool *g_energy*. The interaction energy of the three proteins and of residue Met404, Val425, Ser427, Ala429 and Ala435 for the ligands, were analyzed.

## Results

A preliminary analysis of the available bacterial DnaK sequences was performed to evaluate the reliability of employing the DnaK of *E*.*coli* and *A*.*baumannii* as representative for all the possible bacterial proteins. Moreover, this analysis was useful for verifying residue conservation in positions identified as hot spots at the end of the study. The Hsp70-2 was chosen as a representative for human Hsp70s since it is stress-induced, it localizes in the cytoplasm, and it is one of the most diffused in cells [40].

A comparative analysis was carried out by simulating complexes of the X-ray structures of *E*.*coli* DnaK in closed conformation, with the SBD lid closed over the peptide binding cavity, and the homology model of *A*.*baumannii* DnaK compared to the homology model of the human Hsp70 bound to Api88, drosocin, heliocin, oncocin and pyrrhocoricin. For each of the five inhibitors, closed conformations of both DnaK and Hsp70 in complex with ADP were simulated for 100 ns. Overall, we obtained about 1,3 μs of equilibrated simulation. The equilibrated parts of MD trajectories were evaluated by interaction analysis, conformation clustering, and were employed for the identification of residues belonging to the peptide binding cavity. Computational alanine scanning and per residue binding energy decomposition were carried out to evaluate the role of residues in protein binding. Moreover, multiple alignments were employed to assess conservation of residues, with particular attention to the SBD. At last, identified residues allowed the design of a pharmacophore model, which can be employed in virtual screening studies for the identification of selective and specific DnaK inhibitors.

### SBD residue conservation

The 64 sequences obtained from Uniprot (querying the database for DnaK genes in bacteria, see the Experimental section for details) and the DnaK of *A*.*baumannii* were aligned (S1 Fig) to identify conserved residues. For the same reason, the 13 human Hsp70 sequences were aligned (S2 Fig). DnaK is highly conserved in bacteria, the residue identity is 73% and similarity is about 80% along the whole sequence. Taking into account NBD, conserved residues are distributed mainly outside the secondary structures: for instance, catalytic residues and residues involved in contacts with the nucleotide belong to conserved loops. βSBD displays a high level of residue conservation, compared to αSBD. In detail, NBD displays 20% of fully conserved and about 20% of strongly conserved residues; βSBD displays 22% of fully conserved residues and 26% of residue similarity; on the other hand, only 2% and 5%, respectively, of residues are fully and strongly conserved in αSBD. Similar results were obtained for the human Hsp70 proteins: considering the whole sequence, NBD and βSBD display about 50–55% of residue identity, while only 35% of αSBD residues are strictly conserved. Comparing bacterial DnaK and human Hsp70 proteins, about 45% of residues are strongly conserved along the whole sequence; similar results are displayed for NBD and βSBD (45–40%, respectively); on the other hand, αSBD displays only 18% of conserved residues. Considering only Hsp70-2, the identities increase slightly by, 47, 52 and 58%, respectively, for the whole sequences, NBD and βSBD, while αSBD remains at 20% of residue identity.

Both identities and similarities between sequences analysed in this study have been evaluated. As shown in Table 1, the global similarity between bacterial sequences is about 80%, while between bacterial and human sequences is about 57%. Identity values are about 10% lower than similarity values.

**Table 1:** Identity and similarity percentages of DnaK and Hsp70 protein sequences.

	*A*.*baumannii* DnaK	*E*.*coli* DnaK	hHsp70-2
*A*.*baumannii* DnaK	100% / 100%	70% / 80%	45% / 58%
*E*.*coli* DnaK		100% / 100%	45% / 55%
hHsp70-2			100% / 100%

### Identification of interaction key residues

In order to identify stable DnaK residues anchoring the inhibitor-peptides, the temporal evolution of the interactions was evaluated. The presence of hydrogen bonds and contacts was analysed along the whole MD trajectories. Residues involved in hydrogen bonds with the inhibitor-peptide for at least 25% of the simulation (50% for contacts) were taken into account. Among these residues, only those conserved in DnaK and not in Hsp70 were further considered for identifying selective amino acids for the bacterial protein (Table 2 and Table 3). Four residues were identified as involved in hydrogen bonds: Met404, Ser427, Ala429 and Gln433. The first three residues are substituted by amino acids with different characteristics in Hsp70, respectively Ala404, Thr427 and Tyr429, while Gln433 is maintained in the human protein, but it is rarely involved in hydrogen bonds. Moreover, Met404 and Ala429 hydrogen bonds are conserved in different DnaK complexes, while only two hydrogen bonds were observed in this position for Hsp70 complexes. In detail, the hydrogen bond involving Ser427 is maintained in Hsp70 with a different amino acid, Thr427. In addition to residues involved in hydrogen bonds, Val436 was identified as involved in contacts. Val435 is conserved, but it is rarely involved in contacts in human Hsp70.

**Table 2:** Residues forming hydrogen bonds with the bound peptides.

	*A. baumannii* DnaK					*E. coli* DnaK					hHsp70				
	Api88	droso	helio	onco	pyrr	Api88	droso	helio	onco	pyrr	Api88	droso	helio	onco	pyrr
β1—L_1,2_ - β2	-	-	E402	-	-	-	E402	E402	-	-	-	E402	-	-	-
	-	-	-	-	-	-	T403	-	-	-	-	-	T403	-	T403
	M404	M404	M404	M404	M404	M404	-	M404	M404	M404	A404	A404	-	-	-
	-	-	-	-	-	-	-	-	-	-	G405	-	-	-	-
	-	-	-	-	-	-	-	-	-	-	G406	-	-	-	-
	T409	-	-	-	-	-	-	-	-	-	-	-	T409	-	-
β3—L_3,4_ - β4	-	-	-	-	-	-	-	-	-	-	Q424	-	-	-	-
	-	V425	V425	-	-	-	V425	-	-	-	T425	T425	-	-	-
	S427	S427	S427	S427	S427	S427	S427	S427	S427	S427	T427	T427	T427	T427	T427
	A429	-	A429	A429	A429	A429	A429	-	A429	A429	-	Y429	-	-	Y429
	-	-	-	-	-	-	E430	E430	E430	-	-	-	-	S430	-
	Q433	Q433	Q433	Q433	Q433	Q433	Q433	Q433	Q433	Q433	-	-	-	-	Q433
	-	D437	D437	D437	D437	T437	T437	-	T437	T437	-	-	-	-	L437
HB	-	-	-	-	-	D530	-	-	-	-	-	-	-	-	-
	-	-	-	-	-	-	Q534	-	Q534	Q534	-	-	-	-	-
	-	N537	-	N537	-	-	-	-	-	-	-	-	-	-	-
	-	-	-	E538	-	Q538	-	Q538	Q538	Q538	-	N538	N538	N538	-
	-	D540	D540	-	-	-	-	-	-	-	-	-	-	-	-
	-	-	-	-	-	-	-	-	-	H541	-	-	E541	-	-
	S544	-	-	S544	-	-	-	-	-	-	-	-	-	-	-
	-	-	-	-	-	-	-	-	-	-	-	D580	D580	-	-
	-	-	-	-	Q600	-	-	-	-	-	-	-	-	-	-

Residues involved in interactions conserved in DnaK proteins and not in Hsp70 proteins are underlined.

**Table 3:** Residues involved in contacts with the bound peptides.

	*A. baumannii* DnaK					*E. coli* DnaK					hHsp70				
	Api88	droso	helio	onco	pyrr	Api88	droso	helio	onco	pyrr	Api88	droso	helio	onco	pyrr
β1—L_1,2_ - β2	-	-	-	-	-	-	-	-	-	-	-	E402	-	-	-
	T403	T403	T403	T403	T403	T403	-	T403	T403	T403	T403	T403	T403	T403	T403
	M404	M404	M404	M404	-	M404	M404	M404	-	M404	A404	-	A404	A404	-
β3—L_3,4_ - β4	-	-	-	-	-	-	-	-	-	-	-	-	Q424	-	-
	-	-	V425	-	-	-	-	-	-	-	-	-	-	-	-
	F426	F426	F426	F426	F426	F426	F426 F426	F426	F426	F426	F426	F426	F426	F426	F426
	S427	S427	S427	S427	S427	S427	S427	-	S427	S427	-	T427	-	T427	-
	A429	A429	A429	A429	A429	A429	A429	A429	A429	A429	Y429	Y429	Y429	Y429	Y429
	-	-	-	-	-	-	E430	E430	-	-	-	-	-	-	-
	Q433	-	-	Q433	Q433	Q433	Q433	-	Q433	Q433	-	-	-	-	Q433
	-	-	-	-	A435	A435	-	-	-	A435	-	-	-	-	-
	V436	V436	-	V436	V436	V436	-	-	V436	V436	-	-	-	-	V436
	-	-	D437	D437	D437	-	-	-	T437	-	-	-	-	-	L437
HB	P525	-	-	-	-	-	-	-	-	-	-	-	-	-	-
	-	-	-	-	-	-	Q534	-	Q534	-	-	-	-	-	-
	-	-	-	-	N537	-	-	-	-	N537	-	-	-	-	-
	-	-	-	-	-	-	Q538	Q538	-	-	N538	N538	-	-	-
	-	-	-	-	-	-	-	-	-	-	-	-	-	A539	A539
	-	-	-	D540	-	-	-	-	-	-	-	-	-	-	-
	-	-	-	-	-	H541	-	-	H541	-	-	-	-	-	-
	-	-	-	-	-	-	-	-	-	-	S542	-	-	S542	-
	-	-	-	-	-	-	-	-	-	-	Y543	-	-	-	-
	-	-	-	-	-	-	-	-	-	-	-	-	D580	-	-

Residues involved in interactions conserved in DnaK proteins and not in Hsp70 proteins are underlined.

From the peptide point of view, amino acids involved in interactions with the binding site were analysed. In particular, Met 404 frequently interacts with both side-chain and backbone of residues in the position 4 of peptides, while Ala429 and Gln433 interact with the backbone of residues in positions 5 and 6, respectively. Moreover, Ser427 and Val436 establish contacts with the backbone of residues in positions 2–5 and with the core residue side-chains of the inhibitor-peptides (5 and 6). Concerning residues conservation in peptides, only Proline and Arginine enrichment can be reported.

### Definition of the binding cavity

The binding cavity of DnaK consists of βSBD strands and loops and is closed by the HB helix of αSBD. Residues belonging to the peptide binding cavity were identified from MD simulations of DnaK and Hsp70 bound to the inhibitor-peptides. To this end, the distance matrix between peptide residues and SBD was obtained and residues within 0.5nm from the peptides were considered as pertaining to the binding cavity. Identified residues belong to β_1_—L_1,2_ - β_2_, β_3_—L_3,4_ - β_4_, L_5,6_ - β_6_ and HB (Table 4). All the inhibitors display similar results: differences between DnaK and Hsp70 include Met404, Val425, Ser427, Ala429, Ala435, Ile438 and Gly468 substituted by an Ala, Thr, Thr, Tyr, Ser and Val in the human protein, respectively, while Gly468 (469 in human) is conserved, but point towards the outside of the cavity.

**Table 4:** Binding cavity residues.

	*A*.*baumannii* DnaK	*E*.*coli* DnaK	hHsp70
β1—L_1,2_ - β2	I401, E402, T403, **M404**, G405, G406, V407, L408, T409, P410	I401, E402, T403, **M404**, G405, G406, V407, T409	I401, E402, T403, A404, G405, G406, V407, T409, L411
β3—L_3,4_ - β4	Q424, **V425**, F426, **S427**, T428, **A429**, A430, N432, Q433, P434, **A435**, V436, D437, **I438**, S439, Y441	Q424, **V425**, F426, **S427**, T428, **A429**, E430, D431, N432, Q433, S434, **A435**, V436, T437, **I438**, H439	Q424, T425, F426, T427, T428, Y429, S430, D431, N432, Q433, V436, L437, V438, Q439
L_5,6_ – β6	Q458, L459, R467, **G468**, V469, I472	N458, R467, **G468**, M469, I472, V474	R468 (467), Q473 (471), I474 (472)
HB	N537, D540, A541, S545	D530, E531, Q534, T535, N537, Q538, H541	V536 (533), A537 (534), N540 (537), A541 (538), L542 (539), E543 (540), S544 (541), Y545 (542), Y547 (544), Q596 (593)

Residues conserved in all the three proteins are underlined, while residues conserved only in DnaK are bolded.

### Computational alanine scanning

**Fig 3:**
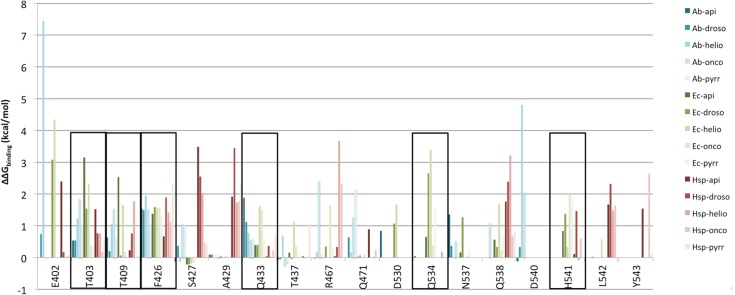
ΔΔGbind results of alanine scanning on binding cavity residues. Interesting residues are displayed for E.coli, A.baumannii DnaK and human Hsp70 in complex with known inhibitors.

Alanine scanning is a computational approach to predict the importance of each residue for a specific protein-protein interface, relying on an energy criterion. Alanine scanning of residues composing the binding cavity was performed on representative conformations achieved through MD simulations of the two DnaK proteins bound to ADP and the inhibitor-peptides (Api88, drosocin, heliocin, oncocin, and pyrrhocoricin). Representative conformations were obtained by cluster analysis, see Experimental section for details. A binding energy threshold (ΔΔGbind) of 1 kcal/mol for residues replaced with alanine was applied to identify residues responsible of protein-protein binding as specified in Kortemme et al. [41]. Residues with a ΔΔGbind value greater than 1 kcal/mol for at least three inhibitor-peptides in both bacterial DnaK proteins were considered. Overall relevant residues are: Thr403, Thr409, Phe426, Gln433, Gln534 and His541 (Fig 3). The first four residues belong to β-SBD and are highly conserved in the bacterial DnaK, while less conserved in the human Hsp70. On the other end, the last two residues belong to the HB helix and are slightly conserved both in bacterial and in human Hsp70 protein family.

### Per residues binding energy decomposition

We evaluated the energetic contribution made by the sum of Lennard-Jones and Coulomb terms to the binding enthalpy for each residue of the protein binding cavity displaying differences between DnaK and Hsp70 (Table 5). For each residue the relative fraction of the total enthalpy was calculated. Residues that mainly contribute to the enthalpy of binding are Met404, Ser427 and Ala429. In particular, Ser427 is a key residue for the interaction of all the analysed ligands bound to the three proteins, both DnaK proteins and hHsp70. On the other hand, Met404 displays a relevant enthalpic contribution only for api88 bound to all proteins, for Drosocin bound to both DnaK proteins and for Pyrrhocoricin bound to E.coli DnaK. Moreover, Ala429 shows a large contribution for all Api88 and Pyrrhocoricin complexes, for hHsp70-Drosocin, for A.baumannii DnaK and for hHsp70-Oncocin.

**Table 5:** Enthalpic contribution to the binding energy.

Complex		Key residues							
		M404	V425	S427	A429	A435	I438	G468	Total
**Api88**	***A*. *baumannii* DnaK**	*-33*,*17* (6%)	-4,71 (1%)	*-45*,*73* (8%)	*-27*,*10* (5%)	-4,78 (1%)	-9,35 (2%)	-2,60 (0%)	-549,58
	***E*. *coli* DnaK**	*-30*,*84* (7%)	-3,48 (1%)	*-43*,*77* (10%)	*-25*,*82* (6%)	-7,97 (2%)	-5,34 (1%)	-2,76 (1%)	-423,19
	**hHsp70**	*-24*,*99* (5%)	*-39*,*02* (8%)	*-45*,*43* (10%)	*-42*,*78* (9%)	-0,18 (0%)	-3,50 (1%)	-1,25 (0%)	-461,28
**droso**	***A*. *baumannii* DnaK**	*-51*,*10* (10%)	-11,54 (2%)	*-39*,*38* (7%)	-20,17 (4%)	-10,94 (2%)	-8,37 (2%)	-1,40 (0%)	-529,30
	***E*. *coli* DnaK**	*-34*,*02* (7%)	-2,43 (0%)	*-48*,*37* (9%)	-14,34 (3%)	-11,01 (2%)	-3,63 (1%)	0,60 (0%)	-513,29
	**hHsp70**	-4,91 (1%)	-12,26 (3%)	*-51*,*83* (11%)	*-49*,*38* (10%)	*-27*,*43* (6%)	1,49 (0%)	-2,74 (1%)	-472,37
**helio**	***A*. *baumannii* DnaK**	-26,25 (4%)	-26,73 (4%)	*-43*,*44* (7%)	-24,01 (4%)	-13,32 (2%)	-3,03 (0%)	1,03 (0%)	-623,49
	***E*. *coli* DnaK**	-8,27 (1%)	-12,82 (2%)	*-43*,*59* (7%)	-24,36 (4%)	-5,93 (1%)	-4,62 (1%)	7,25 (-1%)	-627,85
	**hHsp70**	-12,83 (2%)	-0,22 (0%)	-25,97 (3%)	-30,43 (4%)	-0,08 (0%)	-1,60 (0%)	0,00 (0%)	-819,03
**onco**	***A*. *baumannii* DnaK**	-20,96 (4%)	-12,98 (3%)	*-60*,*14* (12%)	*-24*,*97* (5%)	-6,54 (1%)	-0,76 (0%)	-0,66 (0%)	-485,95
	***E*. *coli* DnaK**	-20,00 (4%)	-11,00 (2%)	*-54*,*98* (12%)	-18,39 (4%)	-8,84 (2%)	0,38 (0%)	-2,89 (1%)	-464,65
	**hHsp70**	-22,42 (4%)	-10,15 (2%)	*-68*,*33* (14%)	*-40*,*30* (8%)	-1,18 (0%)	-8,54 (2%)	-7,33 (1%)	-500,45
**pyrr**	***A*. *baumannii* DnaK**	-19,10 (4%)	-8,29 (2%)	*-52*,*50* (11%)	*-25*,*52* (5%)	-9,45 (2%)	-7,44 (2%)	-6,20 (1%)	-487,61
	***E*. *coli* DnaK**	*-39*,*03* (9%)	-3,07 (1%)	*-40*,*94* (9%)	*-28*,*60* (6%)	-6,98 (2%)	-6,25 (1%)	-2,38 (1%)	-458,42
	**hHsp70**	-6,84 (2%)	-12,61 (3%)	*-44*,*22* (10%)	*-41*,*97* (10%)	-9,49 (2%)	-5,98 (1%)	-0,37 (0%)	-434,77

Energetic contribution to the ligand interaction of residues in the binding cavity. The percentage of each residue contribution to the total enthalpy is indicated in brackets. The more relevant values are highlighted in italic.

### Evaluation of residue conservation

**Fig 4:**
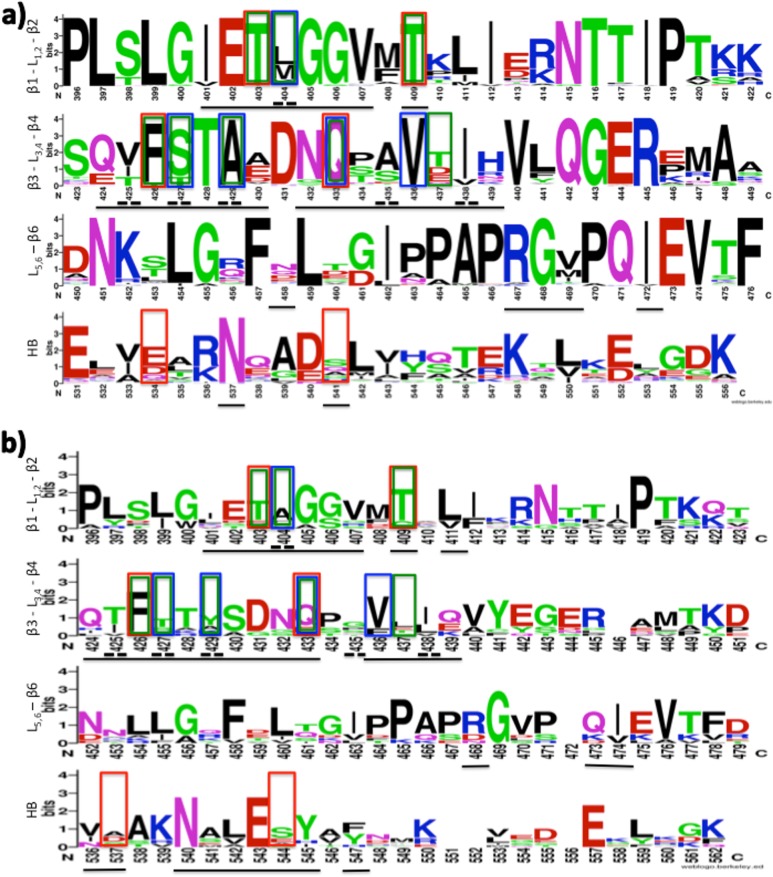
WebLogo representation obtained from the alignment of the 65 DnaK (a) and of the 13 human Hsp70 (b) sequences. Residues belonging to the binding site are underlined, residues identified with alanine scanning analysis are in red boxes, residues obtained by interaction analysis are in blue boxes and residues identified in the pharmacophoric analysis are in green boxes. Residues double underlined are identified hot spots.

At last, the conservation of residues identified with the previous analysis was evaluated. In particular, residue conservation among the 65 DnaK bacterial sequences (Fig 4A) and among the 13 Hsp70 human sequences (Fig 4B) was analysed. Overall, residues belonging to the binding cavities of both proteins are quite conserved. In detail, positions 401–411 (β_1_—L_1,2_ – β_2_) are highly conserved in bacteria, while in human proteins only positions 405, 409 and 411 are well conserved and 402, 403 are quite conserved. Considering residues of β_3_—L_3,4_ – β_4,_ positions 426–429, 431–433, 436, 438 and 440 are highly conserved in bacterial DnaK, while considering human Hsp70 only positions 426, 431 and 436 are conserved. Among residues belonging to L_5,6 –_ β_6_ of DnaK, only positions 458 and 469 are not conserved, while Arg468 and Ile474 are quite conserved also in Hsp70 sequences. Concerning residues of HB, only Glu531, Asn537 and Asp540 (respectively Asn540 and Glu543 for Hsp70) are highly conserved.

### Analysis of structure-based pharmacophoric model

**Fig 5:**
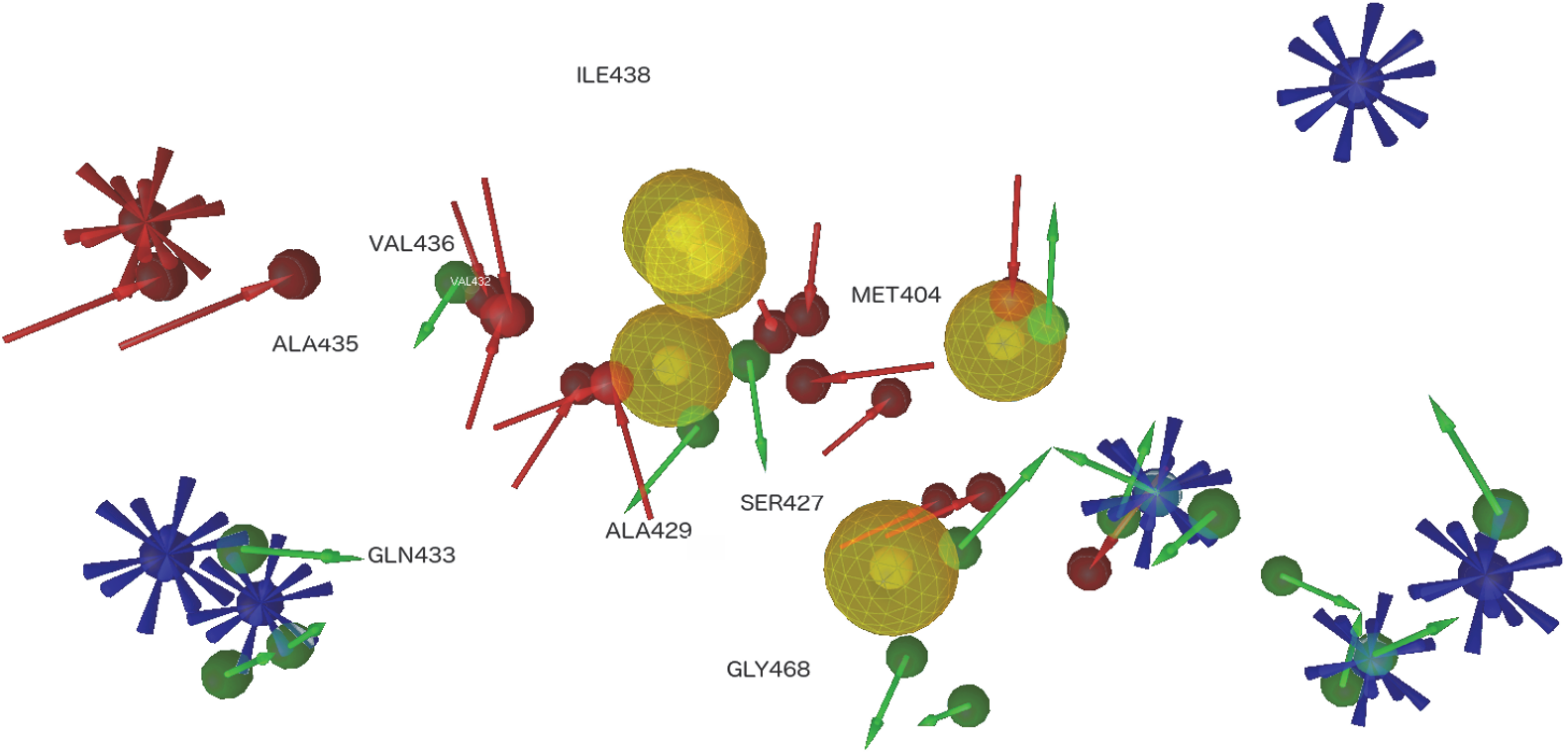
Structure-based pharmacophore. Pharmacophore built on residues important for the interaction between DnaK and inhibitor-peptides and not identified in human proteins. The yellow spheres represent the hydrophobic groups, red and green arrows identify, respectively, H-bonds acceptors and donors. Red and blue spheres are, respectively, negative and positive ionizable groups.

**Fig 6:**
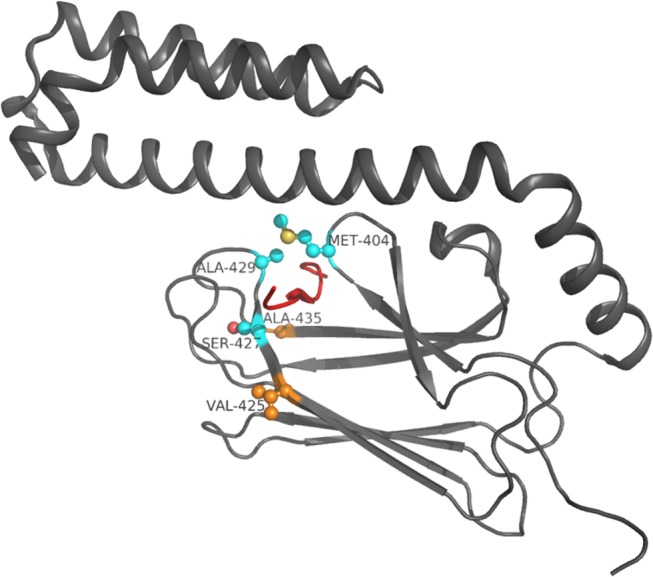
Key residues identified in DnaK SBD. Residue side-chains are in ball and stick, C_α_ atoms of residues previously exploited by known inhibitors are in light blue, C_α_ of the new identified hot spot residues are in orange. The Api88 peptide is represented in red.

The structure-based pharmacophore model comprises the most important pharmacological features for inhibitors binding. Relying on the identified residues, a pharmacophore was obtained for each DnaK-peptide complex (Fig 5). The final pharmacophore, shown in Fig 6, includes the common characteristics of all the models achieved for the different peptides. Several hydrophobic groups (yellow spheres) can be found in the central part of the pharmacophore model. On the other hand, one positive and several negative chargeable groups (red and blue spheres, respectively) localized at both ends of the model. Moreover, along the whole pharmacophore, hydrogen bond donors and acceptors (red and green arrows, respectively) are distributed. This model is in agreement with the inhibitor model identified by Rüdiger et al. [9, 13].

Concerning protein residues, these principally correspond to amino acids identified in previous analysis. In particular, Thr403 and Phe426 are involved only in hydrophobic contacts; Ser427 and Asp/Thr437 are donors or acceptors for hydrogen bonds, while Gln433 results only as an acceptor of hydrogen bond. Moreover, Met404, Thr409 and Ala429 are occupied both in contacts and hydrogen bonds as acceptors or donors.

## Discussion

DnaK is the bacterial homolog of the human Hsp70 and it contributes to bacterial multi-drug resistance, which makes this protein a very interesting drug target. For this reason we propose a detailed study of DnaK binding cavity and its interaction capability in comparison to five recently discovered peptide inhibitors (Api88, drosocin, heliocin, oncocin and pyrrhocoricin) in comparison to the human Hsp70, though structure analysis and molecular dynamics simulations. These methods allow the analysis of complexes in static and dynamic situations. The analysis of known complexes returns residues stably involved in the interaction with inhibitor-peptides, conserved in bacterial DnaK proteins, but not in the human homolog, while more general analyses on the binding cavity residues allow to identify amino acids potentially useful for the design of high affinity and selective ligands.

Overall, residues of βSBD are quite conserved, while residues belonging to helices HA and HB are poorly conserved in bacterial DnaK sequences. Considering only the binding cavity residues, several non-conserved amino acids between bacterial DnaK and human Hsp70 were identified: Met404, Val425, Ser427, Ala429, Ala435, Ile438 and Gly468. These residues are non-conserved in human Hsp70 or are not included in its binding cavity. Our prediction concerning residues, Met404, Ser427, Ala429, Ala435 and Ile438, included in L_3,4_ and L_1,2_ cavity-forming loops, was experimentally confirmed in contacts with the bound peptide [9]. These residues are also involved in the specificity of peptide sequence recognition though van der Waals interactions [9]. Some of these residues were already discussed in the literature: Ile438 was identified among the residues involved in the hydrophobic pocket binding of DnaK [11], while the single mutant S427P displays an ATPase activity similar to the wild type DnaK, but with a weakened peptide binding affinity [42–44]. None of these residues occupy a key position in comparison to the alanine scanning, although Ser427, Ala429, Ile438 and Gly468 result highly conserved in bacterial DnaK proteins, suggesting a selective pressure on these positions. These data were obtained relying on a static conformation, which can explain the different results achieved with our analysis.

Among the identified residues, Met404, Ser427 and Ala429 (Fig 6) are involved in conserved hydrogen bonds and contacts with inhibitor-peptides and are prevalent in the enthalpic contribution to the binding energy. As confirmed by the pharmacophore model, Met404, Ala429 and Ser427 are anchoring points for hydrogen bonds and Met404, Ala429 for hydrophobic contacts, too. Residue Ser427 localized outward the cavity, while Met404 and Ala429 side-chains are closed up to the binding cavity. Looking at these residues, Ser427 (β_3_) and Ala429 (L_3,4_) are highly conserved in bacterial DnaK proteins, while in the human Hsp70 they are substituted by non-conserved residues. On the other hand, Met404 is quite conserved in the bacterial DnaK family and no conserved in the human family. Ala429 forms with Met404 the hydrophobic arch over the peptide backbone [43] and is considered particularly important for the peptide binding [44]. Experimental data confirms that mutations in these positions impair the capability of the proteins to bind peptides [43–45]. Moreover, positions 427 and 429 are included in the binding cavity of Hsp70 with a Thr and a Tyr, respectively.

The binding cavity analysis returned four other residues not involved in the binding of known peptides, which could furthermore be exploited for drug design. Two of these residues, Ile438 and Gly468 are highly conserved both in bacterial DnaK proteins and in the human Hsp70. However Val425 and Ala435 are quite conserved in bacterial, but not in the human protein family, although position 425 presents a conserved Thr residue in human proteins. These two differential conserved residues can be useful anchoring points for the design of selective peptides (Fig 6).

The SBD domain is partially involved in co-chaperones binding, mainly with proteins carrying the J-domain [45]. In particular, the C-terminal helical subdomain is involved in this interaction [46]. Residues identified in this study are not involved in interactions with J-domain proteins.

Our analysis allowed to identify three residues, Met404, Ser427 and Ala429 previously exploited by known inhibitors, and two new anchoring points, Val425 and Ala435, belonging to the binding cavity, probably involved in the inhibition of DnaK binding and selective for bacterial proteins compared to the human Hsp70. In particular, the three known residues localized in the binding cavity anchor the central part of the ligand, while the two new identified residues are on the binding cavity border and can be employed to recognize the external part of the ligand. Moreover, from the binding energy analysis, residues Val425 and Ala435 can be better exploited as anchoring points.

Rational drug design of compounds capable of binding these residues may increase the chance of delivering selective inhibitors. On the other hand, a virtual screening strategy, followed by the selection of ligands that bind critical residues, could also be applied for identifying inhibitors capable of selectively impairing the bacterial DnaK with high affinity.

## Supporting Information

S1 Fig Alignment of bacterial DnaK representative sequences. (PDF)

S2 Fig Human Hsp70 isoforms alignment. (PDF)
