# Supplementary material for: DnaK as Antibiotic Target: Hot Spot Residues Analysis for Differential Inhibition of the Bacterial Protein in Comparison with the Human HSP70
Source: PLoS One. 2015 Apr 23;10(4):e0124563. doi: 10.1371/journal.pone.0124563 (PMC4408060; doi:10.1371/journal.pone.0124563)
Supplement: S1 Fig — (PDF) [file pone.0124563.s001.pdf]

```
Acinetobacter_baumannii          ------MAKIIGIDLGTTNSCVAVLE-GDKVKVIENAEGARTTPSIIAYK-DGEILVGQSAKRQAVTNPKNTLFAIKRLIGRRYEDQAVQKDIG-LVPYK
Agrobacterium_tumefaciens        ------MAKVIGIDLGTTNSCVAVMD-GKDTKVIENAEGARTTPSMVAFSDDGERLVGQPAKRQAVTNPTNTLFAVKRLIGRRYEDPTVEKDKA-LVPFE
Aquifex_aeolicus                 --MAEKKEKIIGIDLGTTNSVVSVMM-GDEAVVIQNQEGSRLTPSVVSWTKEKEILVGEPAKRRAILDPENTVYESKRFIGRKFEE--VKEEAK-RVSYK
Bacillus_anthracis               ------MSKIIGIDLGTTNSCVAVME-GGEPKVIPNPEGNRTTPSVVAFK-NEERQVGEVAKRQAITNPN-TIMSVKRHMGTD-----------------
Bacillus_cereus                  ------MSKIIGIDLGTTNSCVAVME-GGEPKVIPNPEGNRTTPSVVAFK-NEERQVGEVAKRQAITNPN-TIMSVKRHMGTD-----------------
Bacillus_subtilis                ------MSKVIGIDLGTTNSCVAVLE-GGEPKVIANAEGNRTTPSVVAFK-NGERQVGEVAKRQSITNPN-TIMSIKRHMGTD-----------------
Bacteroides_thetaiotaomicron     ------MGKIIGIDLGTTNSCVAVFE-GNEPVVIANSEGKRTTPSVVAFVDGGERKVGDPAKRQAITNPTRTIFSIKRFMGENWDQ--VQKEVT-RVPYK
Bifidobacterium_longum           ------MARAVGIDLGTTNSCIATLE-GGEPTVIVNAEGARTTPSVVAFSKSGEILVGEVAKRQAVTNVDRTISSVKRHMGSD-----------------
Bordetella_pertussis             ------MSKIIGIDLGTTNSCVAVLD-GGQVKIIENAEGARTTPSIVAYMDDGETLVGAPAKRQAVANPKNTLYAVKRLIGRKFDEKAVQKDID-LMPYS
Borrelia_burgdorferi             ------MGKIIGIDLGTTNSCVAIME-HGKPVVIQNSEGGRTTPSIVAYTNKGERLVGQVAKNQMVTNPENTIYSIKRFMGRRFEE--VASEIK-MVPYK
Bradyrhizobium_japonicum         ------MGKVIGIDLGTTNSCVAVMD-GKNAKVIENSEGMRTTPSIVAVTDDGERLVGQPAKRQAVTNPERTFFAVKRLIGRRYDDPMVEKDKK-LVPYK
Buchnera_aphidicola              ------MGKIIGIDLGTTNSCVAIMD-GNKPRVLENAEGDRTTPSIIAYTQEGEVLVGQPAKRQAITNPKNTLFAIKRLIGRKFKDDEVQRDIK-IMPYN
Burkholderia_pseudomallei        ------MGKIIGIDLGTTNSCVAIME-GNQVKVIENSEGARTTPSIIAYMDDNEVLVGAPAKRQSVTNPKNTLFAVKRLIGRRFEEKEVQKDIG-LMPYA
Campylobacter_jejuni             ------MSKVIGIDLGTTNSCVAVYE-RGESKVIPNKEGKNTTPSVVAFTDKGEVLVGDSAKRQAVTNPEKTIYSIKRIMGLMINEDAAKEAKN-RLPYH
Caulobacter_crescentus           ------MSKIIGIDLGTTNSCVAIMD-GKTPKVIENAEGARTTPSVVAFLEDGERLIGQPAKRQAVTNPTNTLFAIKRLIGRTASDPVVEKDKG-MVPYE
Chlamydia_trachomatis            MSEKRKSNKIIGIDLGTTNSCVSVME-GGQPKVIASSEGTRTTPSIVAFK-GGETLVGIPAKRQAVTNPEKTLASTKRFIGRKFSE--VESEIK-TVPYK
Chlorobium_tepidum               ------MGKIIGIDLGTTNSCVAVMQ-GTQPTVIENSEGSRTTPSMVAFTKTGERLVGQAAKRQAVTNPKNTIFSIKRFMGRKYDE--VPNEKK-LASYD
Clostridium_acetobutylicum       ------MSKVIGIDLGTTNSCVAVME-GGDPAVIANSEGARTTPSVVSFQKNGERLVGQVAKRQSITNPDKTIISIKRKMGTA-----------------
Clostridium_botulinum            ------MAKIIGIDLGTTNSCVSVME-GGEPVVIPNAEGSRTTPSVVSFQANGERLIGQVAKRQAITNPEKTIISIKRYMGTD-----------------
Corynebacterium_glutamicum       ------MGRAVGIDLGTTNSVVSVLE-GGEPVVIANAEGSRTTPSVVAFAKNGEVLVGQSAKNQAVTNVDRTIRSVKRHIGTD-----------------
Coxiella_burnetii                ------MAEIIGIDLGTTNSCVAVME-GGKVRVIENAEGSRTTPSIVAYTKDGEVLVGASAKRQAVTNADRTLYAIKRLIGRRFDDNVVQKDIK-MVPYK
Deinococcus_radiodurans          ------MAKAVGIDLGTTNSVIAVME-GGRPEVIVNAEGGRTTPSVVAYK-GDEILVGQIARRQAALNPAATLFEVKRFIGRRWDE--VKEEAA-RSPFT
Desulfovibrio_vulgaris           ------MGKIIGIDLGTTNSCVYVME-GKDPKCITNPEGGRTTPSVVAFTD-KERLVGDIAKRQAVTNPERTVFAVKRLMGRRGDAPEVGRWKE-HSPYR
Dictyoglomus_turgidum            ------MAKIVGIDLGTTNSLIAYLE-GGRPVIIPNAEGSRLTPSIVAFTKDGQLLVGEPAKRQAIVNAERTIRSIKRHMGTN-----------------
Enterococcus_faecalis            ------MSKIIGIDLGTTNSAVAVLE-GGEAKIIANPEGNRTTPSVVSFK-NGEIQVGEVAKRQAVTNPN-TISSIKRHMGEAG----------------
Escherichia_coli                 ------MGKIIGIDLGTTNSCVAIMD-GTTPRVLENAEGDRTTPSIIAYTQDGETLVGQPAKRQAVTNPQNTLFAIKRLIGRRFQDEEVQRDVS-IMPFK
Flavobacterium_psychrophilum     ------MGKIIGIDLGTTNSCVSVME-GQEAVVIPNAEGKRTTPSIIAFVEGGEIKVGDPAKRQAVTNPTKTVASIKRFMGHSFSE--TTDEAK-RVPYS
Francisella_tularensis           ------MGKIIGIDLGTTNSCLAIMD-GKTAKVIENAEGHRTTPSVVAYTDSGEILVGQAAKRQAVTNPDNTFFAIKRLIGRKYDDKAVQEDIKKKVPYA
Fusobacterium_nucleatum          ------MSKIIGIDLGTTNSCVAVME-GGSATIIPNSEGARTTPSVVNIKDNGEVVVGEIAKRQAVTNPTSTVSSIKTHMGSD-----------------
Geobacter_sulfurreducens         ------MSKVIGIDLGTTNSCVAVME-GGEPVVIANAEGSRTTPSMVAFAESGERLVGQQAKRQAVTNPENTLFAIKRLIGRKYDTEEVRKDIS-ISPFK
Gloeobacter_violaceus            ------MGKVVGIDLGTTNSVVAVLE-GGQPTVIANAEGSRTTPSVVAFTKNHDRLVGQLARRQAVLNPENTFYSVKRFIGRKYDE--ITDEAK-QVAYR
Haemophilus_influenzae           ------MGKIIGIDLGTTNSCVAVMD-GDKARVIENAEGARTTPSIIAYT-DNETLVGQPAKRQAITNPKNTLFAIKRLIGRRFESEEVQRDIK-IMPFE
Helicobacter_pylori              ------MGKVIGIDLGTTNSAMAVYE-GNEAKIIANKEGKNTTPSIVAFTDKGEILVGESAKRQAVTNPEKTIYSIKRIMGLMFNEDKAKEAEK-RLPYK
Koribacter_versatilis            ------MAKIIGIDLGTTNSVVAVME-GGEPKVIPNEEGGRTTPSVVGFTKTGERLVGQVAKRQAITNPENTIFSIKRFMGRRYNE--VNEEMK-MVPYK
Lactobacillus_plantarum          ----MASNKIIGIDLGTTNSAVAVLE-GNEPKIITTPEGGRTVPSVVAFK-DGETQVGEVAKRQAITNPN-TVASIKRHMGEAG----------------
Lactococcus_lactis               ------MSKIIGIDLGTTNSAVAVLE-GTESKIIPNPEGNRTTPSVVAFK-NGEIIVGDAAKRQAVTNPE-TIISIKSKMGTS-----------------
Leptospira_interrogans           ----MSKEKIIGIDLGTTNSVVSVME-GGDPVVIQNSEGARTTPSIVAFTAKGETLIGQFAKNQAITNAVNTIRSAKRFIGRRLNE--CESEMK-HVSYK
Listeria_monocytogenes           ------MSKIIGIDLGTTNSAVAVLE-GGEAKIIPNPEGARTTPSVVGFK-NGERQVGEVAKRAAITNPN-TISSIKRHMGTN-----------------
Mesoplasma_florum                ----MAKERIIGIDLGTTNSVVSIME-GGQPIILENPEGQRTTPSVVAFK-NEDIIVGGAAKRQAVTNPN-VVISVKSKMGTN-----------------
Moorella_thermoacetica           ------MSKVIGIDLGTTNSCVAVME-GGEAVVIPNAEGGRTTPSVVAFTKEGERIVGQVAKRQAITNPDRTVISIKRHMGTN-----------------
Mycobacterium_smegmatis          ------MARAVGIDLGTTNSVVAVLE-GGDPVVVANSEGSRTTPSVVAFARNGEVLVGQPAKNQAVTNVDRTIRSVKRHVGTD-----------------
Mycobacterium_tuberculosis       ------MARAVGIDLGTTNSVVSVLE-GGDPVVVANSEGSRTTPSIVAFARNGEVLVGQPAKNQAVTNVDRTVRSVKRHMGSD-----------------
Mycoplasma_mycoides              ----MAKEKIIGIDLGTTNSVVSVIE-GGQPIILENPEGQRTTPSVVAFK-NSDIIVGGAAKRQAVTNPN-VVQSIKSKMGTT-----------------
Mycoplasma_pneumoniae            --MSTDNGLIIGIDLGTTNSCVSVME-NGRPVVLENPEGKRTTPSIVSYK-NNEIIVGDAAKRQMVTNPN-TIVSIKRLMGTSNKV--T-----------
Neisseria_gonorrhoeae            ------MAKVIGIDLGTTNSCLAISE-NGQTKVIENAEGARTTPSIIAYLDGGEILVGAPAKRQAVTNAKNTIYAAKRLIGHKFEDKEVQRDIE-SMPFE
Neisseria_meningitidis           ------MAKVIGIDLGTTNSCLAISE-NGQTKVIENAEGARTTPSVIAYLDGGEILVGAPAKRQAVTNAKNTIYAAKRLIGHKFEDKEVQRDIE-SMPFE
Pseudomonas_aeruginosa           ------MGKIIGIDLGTTNSCVAILE-NGNVKVIENAEGARTTPSIIAYTNDGETLVGQPAKRQAVTNPQNTLYAVKRLIGRRFEENVVQKDIQ-MVPYS
Rhodobacter_sphaeroides          ------MAKVIGIDLGTTNSCVAIMD-GAQPRVIENSEGARTTPSIVGFTD-SERLVGQPAKRQAVTNPSNTVFAVKRLIGRRVGDAEVEKDKK-LVPYS
Rhodopirellula_baltica           ---MAQGEKIIGIDLGTTNSVVAIME-GSEPKVIPNPEGNRLTPSVVAFTDKQETIVGEPARRQAVTNPKRTVYSAKRFMGRRHNE--VQSEEK-MVPYG
Rhodospirillum_rubrum            ------MSKVIGIDLGTTNSCVAVMD-GKDVRVIENAEGARTTPSQVAFTESGERLVGQPAKRQAVTNPENTLFAIKRLIGRRYSDPTVEKDKG-LVPYR
Rickettsia_prowazekii            ------MGKVIGIDLGTTNSCVAVME-GKEPKVIDNAEGERTTPSIIAFAN-SERLVGQPAKRQAVTNPRNTIYAVKRLIGRNFTDPMVRKDQG-LVPYN
Salinibacter_ruber               ------MGKVIGIDLGTTNSVVAVME-GDDPEVIENAEGSRTTPSVVAYKDDGERLVGAPAKRQAITNPENTVSSIKRFMGRFYDE--VEDEIE-EVPYE
Salmonella_typhimurium           ------MGKIIGIDLGTTNSCVAIMD-GTQARVLENAEGDRTTPSIIAYTQDGETLVGQPAKRQAVTNPQNTLFAIKRLIGRRFQDEEVQRDVS-IMPYK
Shewanella_oneidensis            ------MGKIIGIDLGTTNSCVAVLD-GGKARVLENAEGDRTTPSIIAYT-DDETIVGQPAKRQAVTNPNNTFFAIKRLIGRRFKDDEVQRDVN-IMPFK
Shigella_dysenteriae             ------MGKIIGIDLGTTNSCVAIMD-GTTPRVLENAEGDRTTPSIIAYTQDGETLVGQPAKRQAVTNPQNTLFAIKRLIGRRFQDEEVQRDVS-IMPFK
Staphylococcus_aureus            ------MSKIIGIDLGTTNSCVTVLE-GDEPKVIQNPEGSRTTPSVVAFK-NGETQVGEVAKRQAITNPN-TVQSIKRHMGTD-----------------
Streptococcus_pneumoniae         ------MSKIIGIDLGTTNSAVAVLE-GTESKIIANPEGNRTTPSVVSFK-NGEIIVGDAAKRQAVTNPD-TVISIKSKMGTS-----------------
Streptomyces_coelicolor          ------MARAVGIDLGTTNSVVSVLE-GGEPTVITNAEGARTTPSVVAFAKNGEVLVGEVAKRQAVTNVDRTIRSVKRHMGTD-----------------
Synechocystis_sp.                ------MGKVVGIDLGTTNSCVAVME-GGKPTVIANAEGFRTTPSVVGYAKNGDRLVGQIAKRQAVMNPGNTFYSVKRFIGRKFDE--ITNEAT-EVAYS
Thermodesulfovibrio_yellowstonii ------MGKAIGIDLGTTNSVVAVVV-GGEPVVIPNQEGQRTTPSVVAFTDKGERLVGQVAKRQAITNPENTIFSIKRLMGRKYNSQEVQEAKK-RLPYK
Thermotoga_maritima              --MAEKKEFVVGIDLGTTNSVIAWMKPDGTVEVIPNAEGSRVTPSVVAFTKSGEILVGEPAKRQMILNPERTIKSIKRKMGTD-----------------
Thermus_thermophilus             ------MAKAVGIDLGTTNSVIAVLE-GGKPVVLENAEGERVTPSVVAFR-DGETLVGRMAKRQAVLNPEGTIFEIKRFIGRRFEE--VQEEAK-RVPYK
Vibrio_cholerae                  ------MGKIIGIDLGTTNSCVAVLD-GDKPRVIENAEGERTTPSVIAYT-DGETLVGQPAKRQAVTNPQNTLFAIKRLIGRRFEDEEVQRDIK-IMPYK
Xanthomonas_campestris           ------MGKIIGIDLGTTNSCVAIMD-GGKARVIENSEGDRTTPSIVAYTKDGEVLVGASAKRQAVTNPKNTFYAVKRLIGRKFTDGEVQKDIS-HVPYG
Yersinia_pestis                  ------MGKIIGIDLGTTNSCVAIMD-GTKARVLENSEGDRTTPSIIAYTQDGETLVGQPAKRQAVTNPQNTLFAIKRLIGRRFQDEEAQRDKD-IMPYK
                                           :********* :           : . ** . .** :      :  :*  *:.    :   ..   *  :*
```

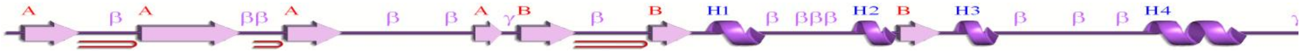

```
Acinetobacter_baumannii            IIKADNGDAW--VEVN-----DKKLAPQQISAEILKKMKKTAEDYLGETVTEAVITVPAYFNDAQRQATKDAGKIAGLDVKRIINEPTAAALAFGMDKK-
Agrobacterium_tumefaciens          IVKGDNGDAW--VKAQ-----DKNYSPSQISAMILQKMKETAESYLGEKVEKAVITVPAYFNDAQRQATKDAGRIAGLDVLRIINEPTAAALAYGLDKK-
Aquifex_aeolicus                   VVPDEKGDAAFDIPNA-----GKLVRPEEVGAHVLRKLKEAAEAFLGEPVKKAVITVPAYFNERQRQATKDAGKIAGLEVVRILNEPTAAAMAYGLHKK-
Bacillus_anthracis                 ----------YKVEVE-----GKDYTPQEISAIILQNLKASAEAYLGETVTKAVITVPAYFNDAERQATKDAGRIAGLEVERIINEPTAAALAYGLEKQ-
Bacillus_cereus                    ----------YKVEVE-----GKDYTPQEISAIILQNLKASAEAYLGETVTKAVITVPAYFNDAERQATKDAGRIAGLEVERIINEPTAAALAYGLEKQ-
Bacillus_subtilis                  ----------YKVEIE-----GKDYTPQEVSAIILQHLKSYAESYLGETVSKAVITVPAYFNDAERQATKDAGKIAGLEVERIINEPTAAALAYGLDKT-
Bacteroides_thetaiotaomicron       VVKGDNNTPR--VDID-----GRLYTPQEISAMILQKMKKTAEDYLGQEVTEAVITVPAYFSDSQRQATKEAGQIAGLEVKRIVNEPTAAALAYGLDKA-
Bifidobacterium_longum             ----------WTVDID-----GKKWTPQEISAQILMKLKRDAEAYLGEPVTDAVITCPAYFNDAQRQATKDAGKIAGLNVLRIINEPTAAALAYGLEKG-
Bordetella_pertussis               IVKADNGDAW--VEVR-----GKKLAPPQVSAEVLRKMKKTAEDYLGEEVTEAVITVPAYFNDSQRQATKDAGRIAGLEVKRIINEPTAAALAFGLDKTE
Borrelia_burgdorferi               IEKGLNGDAR--VNISN---IKKQMSPPEISAATLTKMKETAEAYLGEKVTEAVITVPAYFNDAQRQATKDAGKIAGLEVKRIVNEPTAAALAYGIEKK-
Bradyrhizobium_japonicum           IVKASNGDAW--VEAD-----GQTYSPSQVSAFILQKMKETAEAHLGQKVDQAVITVPAYFNDAQRQATKDAGKIAGLEVLRIINEPTAAALAYGLDKT-
Buchnera_aphidicola                IVNSDNGDAW--IDVK-----KQKMAPPQISAEVLKKMKKTAEDYLGETIKEAVITVPAYFNDAQRQATKDAGRIAGLEVKRIINEPTAAALAYGLDKG-
Burkholderia_pseudomallei          IIKADNGDAW--VEAH-----GEKLAPPQVSAEVLRKMKKTAEDYLGEPVTEAVITVPAYFNDSQRQATKDAGRIAGLEVKRIINEPTAAALAFGLDKAE
Campylobacter_jejuni               ITE-RNGACA--IEIA-----GKIYTPQEISAKVLMKLKEDAEAFLGESVTDAVITVPAYFNDAQRKATKEAGTIAGLNVLRIINEPTSAALAYGLDKKD
Caulobacter_crescentus             IVKGPTGDAW--VKAH-----GKDYSPQEVSAFILQKMKEAAEAHLGEPVTKAVITVPAYFNDAQRQATKDAGKIAGLEVLRIINEPTAAALAYGLDKN-
Chlamydia_trachomatis              VAPNSKGDAV--FDVE-----QKLYTPEEIGAQILMKMKETAEAYLGETVTEAVITVPAYFNDSQRASTKDAGRIAGLDVKRIIPEPTAAALAYGIDKE-
Chlorobium_tepidum                 VVNE-GGYAK--VKIG-----DKTYSPQEISAMILQKMKQTAEDFLGEKVTEAVITVPAYFNDAQRQATKDAGKIAGLEVKRIINEPTAAALAYGLDKK-
Clostridium_acetobutylicum         ----------EKVAID-----DKNYTPQEISAMILQKLKADAEAYLGETVTQAVITVPAYFNDSQRQATKDAGKIAGLEVLRIINEPTAASLAYGLDKM-
Clostridium_botulinum              ----------HKVNID-----STEYTPQQISAMVLQKLKADAEAYLGEKVTQAVITVPAYFNDSQRQATKDAGKIAGLEVLRIINEPTAASLAYGLDKM-
Corynebacterium_glutamicum         ----------WSVAID-----DKNYTSQEISARTLMKLKRDAEAYLGEDVTDAVITVPAYFEDSQRQATKEAGQIAGLNVLRIVNEPTAAALAYGLEKG-
Coxiella_burnetii                  IIKADNGDAW--VEVKDKEGKSQKLAPPQISAQVLIKMKKTAEDYLGHEVKDAVITVPAYFNDSQRQATKDAGKIAGLNVKRIINEPTAAALAYGMDKK-
Deinococcus_radiodurans            VKEGPSGSVR--IEVN-----GKDLAPEQVSAEVLRKLVSDASAKLGNKITDAVITVPAYFDNSQREATRQAGEIAGLNVLRVINEPTAAALAYGLERK-
Desulfovibrio_vulgaris             IVAGANGDAA--VEVQ-----GRPYSAPEISAMILGKLKADAEAYLGETVTEAVITVPAYFNDAQRQATKDAGRIAGLDVKRIINEPTAASLAYGFDRK-
Dictyoglomus_turgidum              ----------YKVKID-----DKEYTPQEISAMILRKLKRDAEAYLGEKIEKAVITVPAYFSDAQRQATKDAGAIAGLEVVRIINEPTAAALAYGLDKE-
Enterococcus_faecalis              ----------YKVDVE-----GKSYTPQEVSAMILQYLKGFAEDYLGEKVEKAVITVPAYFNDAQRQATKDAGKIAGLEVERIVNEPTAAALAYGLDKT-
Escherichia_coli                   IIAADNGDAW--VEVK-----GQKMAPPQISAEVLKKMKKTAEDYLGEPVTEAVITVPAYFNDAQRQATKDAGRIAGLEVKRIINEPTAAALAYGLDKG-
Flavobacterium_psychrophilum       VVKGDNNTPR--VDID-----GRLYTAQELSAMTLQKMKKTAEDYLGQTVTEAVITVPAYFNDAQRQATKEAGEIAGLKVMRIINEPTAAALAYGLDKK-
Francisella_tularensis             VIKADNGDAW--VATK----EGKKMAPPQVSAEVLRKMKKTAEDYLGEPVTEAVITVPAYFNDSQRQATKDAGKIAGLEVKRIINEPTAAALAYGVDSK-
Fusobacterium_nucleatum            ----------YKVEIF-----GKKYTPQEISAKTLQKLKKDAEAYLGEEVKEAVITVPAYFTDSQRQATKDAGTIAGLDVKRIINEPTAAALAYGLEKK-
Geobacter_sulfurreducens           IVKADNGDAW--VEAR-----GKMYSAPEISAMVLQKMKQTAEDYLGETVTDAVITVPAYFNDSQRQATKDAGKIAGLNVLRIINEPTAAALAYGLDKK-
Gloeobacter_violaceus              VVRDGS-NVKLHSTNE-----DKDFAPEEISALVLRKLVDDASKYLGEKITQAVITVPAYFNDSQRQATKDAGRIAGIEVLRIINEPTAAALAYGLDKK-
Haemophilus_influenzae             ITRADNGDAW--VNVK-----GDKLAPPQISAEVLKKMKKTAEDFLGESVTEAVITVPAYFNDAQRQATIDAGKIAGLDVKRIINEPTAAALAFGLGSS-
Helicobacter_pylori                IVD-RNGACA--IEIS-----GKVYTPQEISAKILMKLKEDAESYLGESVTEAVITVPAYFNDSQRKATKEAGTIAGLNVLRIINEPTSAALAYGLDKKE
Koribacter_versatilis              VQQAGD-HVA--VLAQ-----GKEYSPAEISAYILQKLKKAAEDYLGETVTEAVITVPAYFNDAQRQATKDAGKIAGLDVKRIINEPTAAALAYGLDKK-
Lactobacillus_plantarum            ----------YKVSIE-----GKDYTPQQISAMILQYIKGFAEDYLGDTVEKAVVTVPAYFNDAQRQATKDAGKIAGLNIERIINEPTAAALAYGLDKT-
Lactococcus_lactis                 ----------EKVSAN-----GKEYTPQEISAMILQNLKATAEAYLGEKVEKAVITVPAYFNDAQRQATKDAGKIAGLEVERIVNEPTAAALAYGLDKT-
Leptospira_interrogans             VIRSGNEGVK--FETS-----AGEFTPQEISARVLMKMKQTAEDYLGQKVTKAVITVPAYFNDEQRQATKDAGRIAGLEVERIINEPTAAALAYGFDKK-
Listeria_monocytogenes             ----------YKETIE-----GKDYSPQEISAIILQYLKSYAEDYLGETVDKAVITVPAYFNDAQRQATKDAGKIAGLEVERIINEPTAAALAYGMDKT-
Mesoplasma_florum                  ----------EKIDIN-----GKKYTPEQISAEILRYMKKYAEEKVGEKITKAVITVPAYFNDSQRKATKDAGKIAGLDVERIINEPTAAALAYGLEKK-
Moorella_thermoacetica             ----------YKVKID-----NKEYTPQEISAMILQKLKADAEAYLGEKVTQAVITVPAYFTDSQRQATKDAGRIAGLEVLRIINEPTAASLAYGLDKG-
Mycobacterium_smegmatis            ----------WNIEID-----DKKYTPQEISARVLMKLKRDAESYLGEDITDAVITVPAYFNDAQRQATKEAGQIAGLNVLRIVNEPTAAALAYGLDKG-
Mycobacterium_tuberculosis         ----------WSIEID-----GKKYTAPEISARILMKLKRDAEAYLGEDITDAVITTPAYFNDAQRQATKDAGQIAGLNVLRIVNEPTAAALAYGLDKG-
Mycoplasma_mycoides                ----------SKVNLE-----GKDYSPEQISAEILRYMKNYAEAKLGQKVTKAVITVPAYFNDAQRKATKDAGTIAGLQVERIINEPTAAALAYGLDKQ-
Mycoplasma_pneumoniae              -V--------KNPDGS-----TKELTPEEVSAQILSYLKDYAEKKIGKTISRAVITVPAYFNDAERNATKTAGKIAGLNVERIINEPTAAALAYGIDKS-
Neisseria_gonorrhoeae              IIKADNGDAW--VKAQ-----GKELSPPQISAEVLRKMKEAAEAYLGEKVTEAVITVPAYFNDSQRQATKDAGRIAGLDVKRIINEPTAAALAFGMDKGD
Neisseria_meningitidis             IIKANNGDAW--VKAQ-----GKELSPPQISAEVLRKMKEAAEAYLGEKVTEAVITVPAYFNDSQRQATKDAGRIAGLDVKRIINEPTAAALAFGMDKGD
Pseudomonas_aeruginosa             IVKADNGDAW--VEVK-----GQKMAPPQISAEVLKKMKKTAEDYLGEPVTEAVITVPAYFNDSQRQATKDAGRIAGLDVKRIINEPTAAALAYGLDKA-
Rhodobacter_sphaeroides            IVNGGNGDAW--VEVR-----GEKYSPSQISAFILQKMKETAEAYLGESVTQAVITVPAYFNDAQRQATKDAGKIAGLEVLRIINEPTAAALAYGLDKK-
Rhodopirellula_baltica             ITGGPGDY--VKIQVG-----DSEYTPQEISAKVLRKLKESAESYLGHKVNKAVITVPAYFNDAQRQATKDAGQIAGLEVARIINEPTAAALAYGLDKK-
Rhodospirillum_rubrum              IVKGDNGDAW--VQTR-----DEKYAPSQLSAFILQKMKETAEAHLGEPVTQAVITVPAYFNDSQRQATKDAGKIAGLEVLRIINEPTAAALAYGMDKK-
Rickettsia_prowazekii              IVKADNGDAW--VEAD-----NHKYSPSQISAFILQKMKETAENYLGEKVTQAVITVPAYFNDAQRQATKDAGKIAGLEVLRIINEPTAAALAYGFEKS-
Salinibacter_ruber                 VVRGENDTAR--VQIG-----DRKYTPQEISAVVLQKLKQTAEDYLGQEVTDAVITVPAYFNDAQRKATQEAGEIAGLNVQRIINEPTAASLAYGLDDE-
Salmonella_typhimurium             IIGADNGDAW--LDVK-----GQKMAPPQISAEVLKKMKKTAEDYLGEPVTEAVITVPAYFNDAQRQATKDAGRIAGLEVKRIINEPTAAALAYGLDKE-
Shewanella_oneidensis              IIAADNGDAW--VESR-----GNKMAPPQVSAEILKKMKKTAEDFLGEEVTEAVITVPAYFNDSQRQATKDAGRIAGLEVKRIINEPTAAALAYGIDKK-
Shigella_dysenteriae               IIAADNGDAW--VEVK-----GQKMAPPQISAEVLKKMKKTAEDYLGEPVTEAVITVPAYFNDAQRQATKDAGRIAGLEVKRIINEPTAAALAYGLDKG-
Staphylococcus_aureus              ----------YKVDIE-----GKSYTPQEISAMILQNLKNTAESYLGEKVDKAVITVPAYFNDAERQATKDAGKIAGLEVERIINEPTAAALAYGLDKT-
Streptococcus_pneumoniae           ----------EKVSAN-----GKEYTPQEISAMILQYLKGYAEDYLGEKVTKAVITVPAYFNDAQRQATKDAGKIAGLEVERIVNEPTAAALAYGLDKT-
Streptomyces_coelicolor            ----------WKVNLD-----GKDFNPQQISAFVLQKLKRDAESYLGEKVTDAVITVPAYFNDAERQATKEAGEIAGLNVLRIVNEPTAAALAYGLDKD-
Synechocystis_sp.                  VVKDGNGNVKLDCPAQ-----GKQFAPEEISAQVLRKLVDDASKYLGETVTQAVITVPAYFNDSQRQATKDAGKIAGIEVLRIINEPTAASLAYGLDKK-
Thermodesulfovibrio_yellowstonii   IVEAPNGDAH--VEIM-----GKRYSPPEISAMILQKLKQAAEDYLGEPVTEAVITVPAYFDDSQRQATKDAGRIAGLNVLRIINEPTAAALAYGLDKK-
Thermotoga_maritima                ----------YKVRID-----DKEYTPQEISAFILKKLKNDAEAYLGGEIKKAVITCPAYFNDAQRQATKEAGIIAGLEVLRIINEPTAAALAYGLDKA-
Thermus_thermophilus               VVPGPDGGVR--VEVK-----GKLYTPEEISAMILRKLVEDASKKLGEKITKAVITVPAYFNNAQREATANAGRIAGLEVLRIINEPTAAALAYGLDKK-
Vibrio_cholerae                    IVKADNGDAW--VEAK-----GQKMAAPQVSAEVLKKMKKTAEDFLGEPVTAAVITVPAYFNDAQRQATKDAGRIAGLEVKRIINEPTAAALAYGLDKQ-
Xanthomonas_campestris             ILAHDNGDAW--VQTS----DSKRMAPQEISARVLEKMKKTAEDFLGEKVTEAVITVPAYFNDSQRQATKDAGRIAGLDVKRIINEPTAAALAYGLDKN-
Yersinia_pestis                    IIAADNGDAW--LEVK-----GQKMAPPQISAEVLKKMKKTAEDYLGEPVTEAVITVPAYFNDAQRQATKDAGRIAGLEVKRIINEPTAAALAYGLDKE-
                                                               ::.*  *  :   *.  :*  :  **:* **** : :* :*  ** ***:.: *:: ***:*::*:*.
```

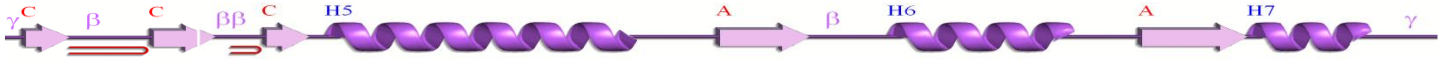

```
Acinetobacter_baumannii          EGDRKVAVYDLGGGTFDVSIIEIADL----DGDQQIEVLSTNGDTFLGGEDFDNALIEYLVEEFKKEQ-NVNLK--NDPLALQRLKEAAEKAKIELSSSN
Agrobacterium_tumefaciens        E-GKTIAVYDLGGGTFDISVLEIG--------DGVFEVKSTNGDTFLGGEDFDMRLVEYLAGEFKKDQ-GIDLK--NDKLALQRLKEAAEKAKIELSSSQ
Aquifex_aeolicus                 -DNVRILVYDFGGGTFDVSILEGGE--------GVIEVKVTAGDTHLGGANIDERIMDWLIEEFKKET-GIDLR--KDRTALQRLKEASEQAKKELSFKM
Bacillus_anthracis               DEEQKILVYDLGGGTFDVSILELAD--------GTFEVISTAGDNRLGGDDFDQVIIDHLVAEFK-KENNIDLS--QDKMALQRLKDAAEKAKKDLSGVT
Bacillus_cereus                  DEEQKILVYDLGGGTFDVSILELAD--------GTFEVISTAGDNRLGGDDFDQVIIDHLVAEFK-KENNIDLS--QDKMALQRLKDAAEKAKKDLSGVT
Bacillus_subtilis                DEDQTILVYDLGGGTFDVSILELGD--------GVFEVRSTAGDNRLGGDDFDQVIIDHLVSEFK-KENGIDLS--KDKMALQRLKDAAEKAKKDLSGVS
Bacteroides_thetaiotaomicron     HKDMKIAVFDLGGGTFDISILEFG--------GGVFEVLSTNGDTHLGGDDFDQVIINWLVQEFKNDE-GADLT--QDPMALQRLKEAAEKAKIELSSST
Bifidobacterium_longum           KEDERILVFDLGGGTFDVSLLEIGKDDD---GFSTIQVQATNGDNHLGGDDWDQKIIDWLVSEVK-NKYGVDLS--KDKIALQRLKEAAEQAKKELSSST
Bordetella_pertussis             KGDRKIVVYDLGGGTFDVSIIEIADV----DGEMQFEVLSTNGDTFLGGEDFDQRIIDYIISEFKKEQ-GVDLS--KDVLALQRLKEAAEKAKIELSSSQ
Borrelia_burgdorferi             H-EEIVAVYDLGGGTFDISILELG--------DGVFEVKSTNGDTHLGGDNFDDEIIKHLISEFKKES-AIDLS--NDKMALQRLKEAAEKAKIELSGAQ
Bradyrhizobium_japonicum         K-TGTIAVYDLGGGTFDISILEIG--------DGVFEVKSTNGDTFLGGEDFDMRLVGYLADEFQKEQ-GINLR--NDKLALQRLKEAAEKAKIELSSTT
Buchnera_aphidicola              KGNRTIAVYDLGGGTFDISIIEIDEV----DKEKTFEVLATNGDTHLGGEDFDSRLINYLVTEFKKEQ-GIDLR--NDPLSMQRLKESAEKAKIELSSAQ
Burkholderia_pseudomallei        KGDRKIAVYDLGGGTFDVSIIEIADV----DGEMQFEVLSTNGDTFLGGEDFDQRIIDYIIGEFKKEQ-GVDLS--KDVLALQRLKEAAEKAKIELSSSQ
Campylobacter_jejuni             --SEKIVVYDLGGGTFDVTVLETG--------DNVVEVLATGGNAFLGGDDFDNKLIDFLANEFKDET-GIDLK--NDVMALQRLKEAAENAKKELSSAN
Caulobacter_crescentus           D-GKKIAVYDLGGGTFDVSILEIG--------DGVFEVKSTNGDTFLGGEDFDLRIVDYLADEFKKEQ-GVDLR--KDKLALQRLREEAEKAKKELSSTA
Chlamydia_trachomatis            -GDKKIAVFDLGGGTFDISILEIG--------DGVFEVLSTNGDTHLGGDDFDGVIINWMLDEFKKQE-GIDLS--KDNMALQRLKDAAEKAKIELSGVS
Chlorobium_tepidum               KENEKVAVFDLGGGTFDISILELG--------GGVFEVKSTDGDTHLGGDDFDQVIINYLADEFKKQE-GIDLR--KDAIALQRLKEAAEKAKIELSSRT
Clostridium_acetobutylicum       DTNQKILVYDLGGGTFDVSVLELGD--------GVFEVKSTNGNTHLGGDDFDEKIMDYIAEEFK-KDNGIDLR--NDKMALQRLKEAAEKAKIELSSST
Clostridium_botulinum            DTNEKILVYDLGGGTFDVSILELGD--------GVFEVKATNGDTKLGGDDFDQKLIDYIAETFK-AENGIDLR--NDKMAIQRLKEAAEKAKIELSSAT
Corynebacterium_glutamicum       EQEQTILVFDLGGGTFDVSLLEIGDG--------VVEVRATSGDNELGGDDWDQRIVDWLVEKFQ-SSNGIDLT--KDKMALQRLREAAEKAKIELSSSQ
Coxiella_burnetii                KGDRKIAVYDLGGGTFDISIIEIAEV----DGEHQFEVLATNGDTFLGGEDFDLRLIDYLAGEFKKDE-GVDLH--NDPLALQRLKEAAEKAKIELSSSQ
Deinococcus_radiodurans          -GNETVLVFDLGGGTFDVTILELGD--------GVFEVKSTAGDTHLGGADFDYRIVDWLAGEFQKEH-NFDLR--KDKQALQRLIEAAEKAKIDLSNAS
Desulfovibrio_vulgaris           A-NEKIAVFDLGGGTFDISILEVG--------DNVVEVRATNGDTFLGGEDFDQRIISYLVDEFRRENGGIDLA--RDRMALQRLKEAAEKAKKDLSTSM
Dictyoglomus_turgidum            G-HQKILVFDLGGGTFDVSILEIGE--------GVFEVIATAGNNRLGGDDFDERIVNWLIENFM-EEHGINLR--EDKTALQRLYEAAEKAKIELSSKL
Enterococcus_faecalis            DKDEKILVFDLGGGTFDVSILELGD--------GVFDVLSTAGDNNLGGDDFDNKIIDYMVAEFK-KENGIDLA--NDKMALQRLKDAAEKAKKDLSGVT
Escherichia_coli                 TGNRTIAVYDLGGGTFDISIIEIDEV----DGEKTFEVLATNGDTHLGGEDFDSRLINYLVEEFKKDQ-GIDLR--NDPLAMQRLKEAAEKAKIELSSAQ
Flavobacterium_psychrophilum     GIDQKIAVYDLGGGTFDISILELG--------DGVFEVLSTNGDTHLGGDDFDQTIIDWLADEFKAEE-GIDLR--LDPMSLQRIKEAAEKAKIELSSSA
Francisella_tularensis           KGEQTVAVYDLGGGTFDISIIEIADV----DGDNQIEVLSTNGDTFLGGEDFDLALMNYLIDEFKKEQ-GIDLH--NDKLALQRVREAAEKAKVELSSAQ
Fusobacterium_nucleatum          K-EEKVLVFDLGGGTFDVSVLEISD--------GVIEVISTAGNNHLGGDDFDNEIINWLVAEFK-KETGIDLS--NDKMAYQRLKDAAEKAKKELSTLM
Geobacter_sulfurreducens         K-DEKIAVFDLGGGTFDISILELG--------DGVFEVKSTNGDTFLGGEDFDQRVIDWIADEFKKDQ-GIDLR--GDKMALQRLKEAAEKAKCELSTSM
Gloeobacter_violaceus            A-NETILVFDLGGGTFDVSILEVGD--------GVFEVKSTSGDTHLGGDDFDRRIVDYLADEFK-KLEGVDLR--TDRQALQRLTEAAEKAKIELSGVT
Haemophilus_influenzae           KENQVIAVYDLGGGTFDISIIEIDNF----DGEQTFEVLATGGNTHLGGEDFDNRVIDYIIDEFKKEQ-NIDLR--NDAMALQRVKEAAEKAKIELSSAQ
Helicobacter_pylori              --SEKIMVYDLGGGTFDVTVLETG--------DNVVEVLATGGDAFLGGDDFDNRVIDFLASEFKSET-GIEIK--NDVMALQRLKEAAENAKKELSSAM
Koribacter_versatilis            K-DETIAVYDFGGGTFDISILEVG--------EGVIEVKSTNGDTHLGGDNIDQRIVEWLIDEFKKKE-GLDLRAKGNEMALQRLRDGAERAKIELSTTM
Lactobacillus_plantarum          DKDEKILVYDLGGGTFDVSILELGD--------GVFEVLSTNGDTHLGGDDFDQKIIDWLVDGFK-ADNGVDLS--KDKMALQRLKDAAEKAKKDLSGVS
Lactococcus_lactis               DKDEKILVFDLGGGTFDVSILELGD--------GVFDVLATAGNNKLGGDDFDQKIIDWMVAEFK-KENGIDLA--QDKMALQRLKDAAEKAKKDLSGVT
Leptospira_interrogans           NVNSKIAVYDLGGGTFDISILELA--------DGVFEVKSTNGDTHLGGDDFDMAIMEWMISEFKNQT-GIDIS--ADKNTVQRLKEAAEKAKIELSGTM
Listeria_monocytogenes           ETDQTILVFDLGGGTFDVSILELGD--------GVFEVHSTAGDNELGGDDFDKKIIDYLVAEFK-KDNGIDLS--QDKMALQRLKDAAEKAKKDLSGVT
Mesoplasma_florum                DKEEKVLVYDLGGGTFDVSILELAD--------GTFEVLSTSGDNKLGGDNFDTQIINWLIEKIK-TESGVDLK--NDKMALQRLKDEAEKAKINLSSQL
Moorella_thermoacetica           E-DQTILVYDLGGGTFDVSILELGD--------GVFEVKATSGNNRLGGDDFDQRIMDYLVDICR-REHGVDLT--QDKMAMQRLKEAAEKAKIELSGMT
Mycobacterium_smegmatis          EKEQTILVFDLGGGTFDVSLLEIGDG--------VVEVRATSGDNHLGGDDWDDRIVTWLVDKFK-GSSGIDLT--KDKMAMQRLREAAEKAKIELSSSQ
Mycobacterium_tuberculosis       EKEQRILVFDLGGGTFDVSLLEIGEG--------VVEVRATSGDNHLGGDDWDQRVVDWLVDKFK-GTSGIDLT--KDKMAMQRLREAAEKAKIELSSSQ
Mycoplasma_mycoides              DKEETILVYDLGGGTFDVSILAIGG--------GSFDVIATSGNNKLGGDNFDEEIIKWLLGKIK-AEYNIDLS--KEKMALQRLKDEAEKAKINLSSQL
Mycoplasma_pneumoniae            NREMKVLVYDLGGGTFDVSLLDIAE--------GTFEVLATAGDNRLGGDDWDNKIIEFILAHIAQEHNGLNLS--NDKMAMQRLKEAAERAKIELSAQL
Neisseria_gonorrhoeae            NKDRKIAVYDLGGGTFDISIIEIANL----DGDKQFEVLATNGDTFLGGEDFDQRLIDYIIDEFKKEQ-GIDLK--QDVMALQRLKEAAEKAKIELSSGQ
Neisseria_meningitidis           NKDRKVAVYDLGGGTFDISIIEIANL----DGDKQFEVLATNGDTFLGGEDFDQRLIDHIIAEFKKEQ-GIDLK--QDVMALQRLKEAAEKAKIELSSGQ
Pseudomonas_aeruginosa           KGDHTVIVYDLGGGTFDVSVIEIAEV----DGEHQFEVLATNGDTFLGGEDFDIRLIDYLVDEFKKES-GINLK--GDPLAMQRLKEAAEKAKIELSSTQ
Rhodobacter_sphaeroides          D-TKTIAVYDLGGGTFDITILEID--------DGLFEVKSTNGDTFLGGEDFDMRIVNYLADEFKKEH-GVDLT--LDKMALQRLKEAAEKAKIELSSSQ
Rhodopirellula_baltica           K-DESIIVFDLGGGTFDVSVLEVADSGDEEQESRVFQVVSTSGDTHLGGDDFDEALINYVASEFQ-KDNGIDLR--NDAMALQRLQEACEKAKKELSTLP
Rhodospirillum_rubrum            N-TGTIAVFDLGGGTFDVSVLEIG--------DGVFEVKSTNGDTFLGGEDFDARIIDYLASEFKKEQ-GIDLR--TDRLALQRLKEAAEKAKIELSSSM
Rickettsia_prowazekii            S-SKTIAVYDLGGGTFDVSILEIS--------DGVFEVKSTNGDTFLGGEDFDTRILNHLIEVFKKES-GIDLS--KDPLALQRLKEAAEKAKKELSSTS
Salinibacter_ruber               -SDQVVAVYDLGGGTFDVSILELG--------DGVFEVNATYGDTHLGGDNFDKRLIDHIADEFEQDT-GIDLR--DDPMALQRLKEAAEEAKIELSSAK
Salmonella_typhimurium           VGNRTIAVYDLGGGTFDISIIEIDEV----DGEKTFEVLATNGDTHLGGEDFDTRLINYLVDEFKKDQ-GIDLR--NDPLAMQRLKEAAEKAKIELSSAQ
Shewanella_oneidensis            QGDNIVAVYDLGGGTFDISIIEIDSN----DGDQTFEVLATNGDTHLGGEDFDNRLINYLADEFKKEQ-GLDLR--KDPLAMQRLKEAAEKAKIELSSTN
Shigella_dysenteriae             TGNRTIAVYDLGGGTFDISIIEIDEV----DGEKTFEVLATNGDTHLGGEDFDSRLINYLVEEFKKDQ-GIDLR--NDPLAMQRLKEAAEKAKIELSSAQ
Staphylococcus_aureus            DKDEKVLVFDLGGGTFDVSILELGD--------GVFEVLSTAGDNKLGGDDFDQVIIDYLVAEFK-KENGVDLS--QDKMALQRLKDAAEKAKKDLSGVS
Streptococcus_pneumoniae         DKEEKILVFDLGGGTFDVSILELGD--------GVFDVLSTAGDNKLGGDDFDQKIIDHLVAEFK-KENGIDLS--TDKMAMQRLKDAAEKAKKDLSGVT
Streptomyces_coelicolor          --EQVILVFDLGGGTFDVSLLEIGDG--------VVEVKATNGDNNLGGDDWDQRVVDYLVKQFQ-SGHGVDLA--KDKMALQRLREAAEKAKIELSSST
Synechocystis_sp.                D-NETILVFDLGGGTFDVSILEVGE--------GVFEVLATSGDTHLGGDDFDKKIVDFLAGEFQ-KAEGIDLR--KDKQALQRLTEAAEKAKIELSGVS
Thermodesulfovibrio_yellowstonii K-EEKIAVYDLGGGTFDISILEIG--------EGVIEVKATNGDTYLGGDDFDIRVMDWLIEEFKKQE-GIDLR--KDRMALQRLKEAAERAKIELSSAM
Thermotoga_maritima              GKEEKVLVYDLGGGTFDVSILEIGE--------GVIEVIATAGNNHLGGDDFDQRLIDWMAEEFK-KQHGIDLR--EDRQALQRLRDAAEKAKIELSTKM
Thermus_thermophilus             -GNETVLVFDLGGGTFDVTILEIGE--------GVFEVKATSGDTHLGGSDMDHAIVNWLAEEFKKEH-GVDLK--ADRQALQRLIEAAEKAKIELSSTL
Vibrio_cholerae                  GGDRTIAVYDLGGGTFDISIIEIDEV----EGEKTFEVLSTNGDTHLGGEDFDNRMINYLVDEFKKDQ-GIDLR--NDPLAMQRLKEAAEKAKIELSSAQ
Xanthomonas_campestris           GGDRKIAVYDLGGGTFDVSIIEIAEV----DGEKQFEVLATNGDTFLGGEDFDNRVIEYLVDEFNKDQ-GIDLR--KDPLALQRLKDAAERAKIELSTSQ
Yersinia_pestis                  VGNRTIAVYDLGGGTFDISIIEIDEV----DGEKTFEVLATNGDTHLGGEDFDSRLINYLVEEFKKDQ-GMDLR--TDPLAMQRLKEAAEKAKIELSSAQ
                                      : *:*:******::::              .:*  * *:  *** : *  ::  :           ::    :  : **: : .*.** :**
```

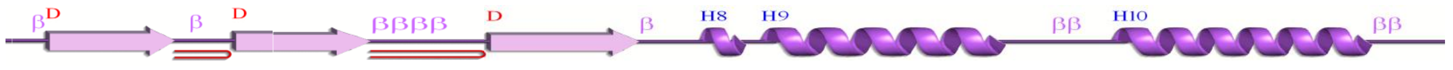

```
Acinetobacter_baumannii           ATEINLPYITADA--TG-PKHLVINVTRAKLEGLVADLVARTIEPCKIALKDAGLSTSDISDVILVGGQSRMPLVQQKVQEFF-GREPRKDVNPDEAVAI
Agrobacterium_tumefaciens         QTEINLPFITADA--SG-PKHLTLKLTRAKFESLVDDLVQRTVAPCKAALKDAGVTAAEIDEVVLVGGMSRMPKVQEVVKQLF-GKEPHKGVNPDEVVAM
Aquifex_aeolicus                  ETEINLPFITIDPNTNQ-PLHLQKKLTRARLEEMIKDIVDRTIDIVKQALEDAKLKPSDIDEVVLVGGSTRIPLVQQRIKEFF-GKEPHKGLNPDEVVAM
Bacillus_anthracis                QTQISLPFISAGA--AG-PLHLELTLTRAKFEELSAGLVERTLEPTRRALKDAGFAPSELDKVILVGGSTRIPAVQEAIKRET-GKEPYKGVNPDEVVAL
Bacillus_cereus                   QTQISLPFISAGA--AG-PLHLELTLTRAKFEELSAGLVERTLEPTRRALKDAGFAPSELDKVILVGGSTRIPAVQEAIKRET-GKEPYKGVNPDEVVAL
Bacillus_subtilis                 STQISLPFITAGE--AG-PLHLELTLTRAKFEELSSHLVERTMGPVRQALQDAGLSASEIDKVILVGGSTRIPAVQEAIKKET-GKEAHKGVNPDEVVAL
Bacteroides_thetaiotaomicron      STEINLPYIMPVN--GV-PKHLVKTLTRAKFESLAHGLIQACLEPCKKAMSDAGLSNSDIDEVILVGGSSRIPAVQKLVEDFF-GKTPSKGVNPDEVVAV
Bifidobacterium_longum            STSISMQYLAMTP--DGTPVHLDETLTRAHFEEMTSDLLGRCRTPFNNVLHDAGISVSDIDHVVLVGGSTRMPAVKELVKELTGGKEANQSVNPDEVVAV
Bordetella_pertussis              QTEINLPYITADA--SG-PKHLNLKITRAKLEALVEELIERTIEPCRVAIKDAGVKVSDIDDVILVGGMTRMPKVQDKVKEFF-GREPRKDVNPDEAVAA
Borrelia_burgdorferi              EASINLPFITADA--NG-PKHLQYTLTRAKFEQMVDHLVQKTKEPCLKAIKDAGLKASDINEVILVGGSTRIPAIQKIVKDIF-GQDPNKGVNPDEAVAI
Bradyrhizobium_japonicum          QTEINLPFITADQ--TG-PKHLTMKLTRAKFEALVDDLIQKTVEPCRKALKDAGVTAGEIGEVVLVGGMSRMPKVQEVVKQLF-GKEPHKGVNPDEVVAI
Buchnera_aphidicola               QTDVNLPYITADS--NG-PKHLNIKVTRAKLESLVEDLILRSIEPLKVALKDAGLSVTDINDVILVGGQTRMPMVQSKVADFF-GKEPRKDVNPDEAVAV
Burkholderia_pseudomallei         QTEINLPYITADA--SG-PKHLNLKVTRAKLEALVEDLVERTIEPCRTAIKDAGVKVSDIDDVILVGGQTRMPKVQEKVKEFF-GKEPRRDVNPDEAVAV
Campylobacter_jejuni              ETEINLPFITADA--SG-PKHLVKKLTRAKFEGMIDSLVAETITKINEVVSDAGLKKDEIKEIVMVGGSTRVPLVQEEVKKAF-NKDLNKSVNPDEVVAI
Caulobacter_crescentus            QYEVNLPFISMNA--SG-PLHLNIKLSRAKLEALVDDLIARTIGPCEQALKDAGLKKSDIDEVILVGGMSRMPKVQQAVQDFF-GREPHKGVNPDEVVAL
Chlamydia_trachomatis             STEINQPFITIDA--NG-PKHLALTLTRAQFEHLASSLIERTKQPCAQALKDAKLSASDIDDVLLVGGMSRMPAVQAVVKEIF-GKEPNKGVNPDEVVAI
Chlorobium_tepidum                DTEINLPFITATQ--EG-PKHLVINLTRAKFEAMSAALFDKLFEPCRRAIKNSKFDIKEIDEVVLVGGSTRIPKVQALVKEFF-GKEPNKSVNPDEVVAI
Clostridium_acetobutylicum        QTNINLPFITADA--TG-PKHIDMNLTRAKFNELTEGLVQDTIEPMKKALSDAGLSINDIDKIVLVGGSTRIPAVQEAVKNYT-GKDPSKGVNPDECVAI
Clostridium_botulinum             QTNINLPFITADA--TG-PKHIDMNLTRAKFNELTHDLVQRTLEPIKKSLEGSGYAMSDIDKIIMVGGSTRIPAVQDAVKDFT-GKELSKGVNPDEVVAM
Corynebacterium_glutamicum        SANINLPYITVDA--DKNPLFLDETLSRAEFQRITQDLLARTKTPFNQVVKDAGVSVSEIDHVVLVGGSTRMPAVTELVKELTGGREPNKGVNPDEVVAV
Coxiella_burnetii                 QTDVNLPYITADA--SG-PKHLNIRLTRAKLESLVEDLVERTIEPCKVAIKDAGLKVSEIDDVILVGGQTRMPKVQEAVKNFF-GKEARKDVNPDEAVAI
Deinococcus_radiodurans           ETSISLPFITFDPETRT-PMHLERSLSRAKFEELTADLLRRVRQPVEQALSDAKLSAGDINEVILVGGSTRIPAVKRIVQDLV-GKTPNESVNPDEAVAL
Desulfovibrio_vulgaris            ETEVNLPFITADQ--TG-PKHLMMKLSRAKLEKLVEDLVERTVEPCRKALADAGLTAAQIDEVVLVGGMTRMPLVQKRVSEFF-GKEPNRSVNPDEVVAM
Dictyoglomus_turgidum             QTEINLPFIAMKG--NT-PLHLSYTLTRAKFEELTYDLVEKTKEPTERALKDAGLSPSQIDKIILVGGATRMPCIQEWIKKHF-GKEPQRNVNPDEAVAL
Enterococcus_faecalis             STQISLPFITAGE--AG-PLHLEMNLTRAKFDELTSDLVERTKVPVRQALKDAGLNPSEIDEVILVGGSTRIPAVVEAVRKET-NKEPNKSVNPDEVVAM
Escherichia_coli                  QTDVNLPYITADA--TG-PKHMNIKVTRAKLESLVEDLVNRSIEPLKVALQDAGLSVSDIDDVILVGGQTRMPMVQKKVAEFF-GKEPRKDVNPDEAVAI
Flavobacterium_psychrophilum      ETEINLPYVTATA--SG-PKHLVKKLTRAKFEQLSDTLVKRSMEPVAKALKDAGLTVKDIDEVILVGGSTRMPRIADEVEKFF-GKKASKGVNPDEVVAI
Francisella_tularensis            QTDVNLPYITADA--TG-PKHLNIKVTRAKFESLVSDLVMRSLEPCKKALEDAGLSKSDITEVLLVGGQTRMPLVQEKVKEFF-GKEPRKDVNPDEAVAV
Fusobacterium_nucleatum           ETSISLPFITMDA--TG-PKHLEMKLTRAKFNDLTKHLVEATQGPTKTALKDASLEANQIDEILLVGGSTRIPAVQEWVENFF-GKKPNKGINPDEVVAA
Geobacter_sulfurreducens          ETDINLPFITADA--TG-PKHLTMKLSRAKLEALCADLLNKLEGPCRTALKDAGLSPSEVDEVILVGGMTRMPAVQKRVQEIF-GKVPNKGVNPDEVVAI
Gloeobacter_violaceus             QTQINLPFITAGA--DG-AKHLDMSLTRAKFESLCADLLRRVEKPVEQALRDAKLSKENIDEVVLVGGSTRIPAVQELVKRII-GKDPNQSVNPDEVVAV
Haemophilus_influenzae            STEVNLPYITADA--TG-PKHLALNITRAKLEALVEDLVASSIESLKAVLKDADKGVSEIHDIILVGGQTRMPLVQQKVAEFF-GKEARKDVNPDEAVAI
Helicobacter_pylori               ETEINLPFITADA--TG-PKHLVKKLTRAKFESLTEDLMKETISKIESVIKDAGLTKNEISEVVMVGGSTRIPKVQERVKAFI-NKDLNKSVNPDEVVAV
Koribacter_versatilis             ETEINLPFITADA--SG-PKHLVERLTRSKLEEMVRDLVDRSIEPCRQALKDAGIDASKIDEVVLVGGQTRMPRIQQVVKEFF-GREPHRGVNPDEVVAI
Lactobacillus_plantarum           EAQISLPFISAGA--SG-PLHLETTLTRAKFNELTADLVEKTRIPVENALKDADLSASDLDVVILNGGSTRIPAVQEAVEKWT-GKESNHSINPDEAVAL
Lactococcus_lactis                TTQISLPFITAGA--AG-PLHLEMALTRAKFDELTHDLVEATRQPVRQALSDAGLSTSDIDEVLLVGGSTRIPAVVELVRHET-NKEPNKSVNPDEVVAM
Leptospira_interrogans            STQINLPFITADA--SG-PKHLDMTLTRAKFDQLTKSLVDRTRIPCENALRDAGLKASDINEVILVGGSIRIPAVQELVKQVF-GKEPNKSVNPDEVVAI
Listeria_monocytogenes            STQISLPFITAGE--AG-PLHLEVTLTRAKFDELTHDLVERTIAPTRQALKDANLSASDIDQVILVGGSTRIPAVQETIKKEL-GKEPHKGVNPDEVVAM
Mesoplasma_florum                 EVEINLPFIAMNE--NG-PVSFSTQFSRTEFDKITKDLVERTSKPVKDALQAAKLSASDIDEVLLVGGSTRIPAVQKIVKELL-GKEPNRSINPDEVVAM
Moorella_thermoacetica            STNINLPFISATP--NG-PVHLDVNLTRAKFEELIADLVEKTVGPTRQALADAGLEPKDIDKVLLVGGSTRVPLVQETVRKIL-GQEPHKGINPDECVAL
Mycobacterium_smegmatis           STSINLPYITVDA--DKNPLFLDEQLTRAEFQRITQDLLDRTRQPFQQVIKDAGISVSDIDHVVLVGGSTRMPAVTDLVKELTGGKEPNKGVNPDEVVAV
Mycobacterium_tuberculosis        STSINLPYITVDA--DKNPLFLDEQLTRAEFQRITQDLLDRTRKPFQSVIADTGISVSEIDHVVLVGGSTRMPAVTDLVKELTGGKEPNKGVNPDEVVAV
Mycoplasma_mycoides               EVEINLPFIAMNE--SG-PISFATTLTRSEFNKITKHLVDLTIQPVKDALSAAKKTPSEINEVLLVGGSTRIPAVQELVKSLL-NKEPNRSINPDEVVAM
Mycoplasma_pneumoniae             EAIISLPFLTVTE--KG-PVNVELKLTRAKFEEITKQLLERTRNPISDVLREAKIKPEEINEILLVGGSTRMPAVQKLVESMVPGHSPNRSINPDEVVAI
Neisseria_gonorrhoeae             QTEINLPYITMDA--TG-PKHLAMKITRAKFESLVEDLIARSIEPCRTALKDAGLSTGDIDDVILVGGQSRMPKVQEAVKDFF-GKEPRKDVNPDEAVAV
Neisseria_meningitidis            QTEINLPYITMDA--TG-PKHLAMKITRAKFESLVEDLITRSIEPCKIALKDAGLSTGDIDDVILVGGQSRMPKVQEAVKAFF-GKEPRKDVNPDEAVAV
Pseudomonas_aeruginosa            QTDVNLPYVTADA--SG-PKHLNVKVSRAKLESLVEDLVQRTIEPCRTALKDAGLDVSDIHEVILVGGQTRMPLVQKTVAEFF-GKEARKDVNPDEAVAV
Rhodobacter_sphaeroides           QTEINQPFISMDRNTGQ-PLHMVMKLTRAKLESLVGDLIKKSLKPCEAALKDAGVSKSDIDEVVLVGGMTRMPRVVEEVTKFF-GKEPHKGVNPDEVVAL
Rhodopirellula_baltica            ETDINLPFITMDA--SG-PKHLTMKITRSKFEELIDALVERCRGPVLQALKDAGMDPKDIDEVVLVGGSTRVPKVREVVKSIF-GKDPHQGVNPDEVVAV
Rhodospirillum_rubrum             QTEVNLPFITADQ--AG-PKHLNIKLTRAKLEALVDDLVQRTVEPCRKALADAGIKASEIDEVILVGGMTRMPKVQQVVKDFF-GREPHKGVNPDEVVAM
Rickettsia_prowazekii             TTDINLPYITADS--TG-PKHLNIKFTRAELEKLVDDLIEKTIEPCRQALKDAGFKPNDIQEVVLVGGMTRMPKVQEAVKKFF-GREPHKGVNPDEVVAL
Salinibacter_ruber                TTTINLPFITATD--EG-PQHLNMDLNRATFENLIEDLVEKTVPQMEKALDDAGHSKSDVDEVILVGGSTRVPLVQETVEDFF-GKQANKSVNPDEVVSL
Salmonella_typhimurium            QTDVNLPYITADA--TG-PKHMNIKVTRAKLESLVEDLVNRSIEPLKVALQDAGLSVSDINDVILVGGQTRMPMVQKKVAEFF-GKEPRKDVNPDEAVAI
Shewanella_oneidensis             QTEVNLPYITADA--TG-PKHLVVKITRAKLESLVEDLIIRTLEPLKVALADADLSVSDINEVILVGGQTRMPKVQEAVTNFF-GKEPRKDVNPDEAVAV
Shigella_dysenteriae              QTDVNLPYITADA--TG-PKHMNIKVTRAKLESLVEDLVNRSIEPLKVALQDAGLSVSDIDDVILVGGQTRMPMVQKKVAEFF-GKEPRKDVNPDEAVAI
Staphylococcus_aureus             QTQISLPFISAGE--NG-PLHLEVNLTRSKFEELSDSLIRRTMEPTRQAMKDAGLTNSDIDEVILVGGSTRIPAVQEAVKKEI-GKEPNKGVNPDEVVAM
Streptococcus_pneumoniae          STQISLPFITAGE--AG-PLHLEMTLTRAKFDDLTRDLVERTKVPVRQALSDAGLSLSEIDEVILVGGSTRIPAVVEAVKAET-GKEPNKSVNPDEVVAM
Streptomyces_coelicolor           ETTINLPYITASA---EGPLHLDEKLTRAQFQQLTSDLLERCKTPFHNVIKDAGIQLSEIDHVVLVGGSTRMPAVAELVKELTGGKDANKGVNPDEVVAI
Synechocystis_sp.                 QTEINLPFITATQ--DG-PKHLDTTLSRAKFEEICSDLIDRCGIPVENAIRDAKIDKSALDEIVLVGGSTRIPAVQEVVKKIL-GKDPNQGVNPDEVVAV
Thermodesulfovibrio_yellowstonii  ETEINLPFITADA--SG-PKHLLMKLTRAKLEQLVDDLIQKSLEPCKKALSDAGLSQSQIDEVILVGGQTRTPKVQKVVQDFF-GKEPHKGVNPDEVVAV
Thermotoga_maritima               ETDVSLPFIAVSP--SGQPLHLEMRITRSLFESLTRDLVEMTRGPIEQALNDAKLSPQDIDEIILVGGMTRVPMVQRFIKEFF-GKEPNKSVNPDEAVAI
Thermus_thermophilus              ETTISLPFIALDPASKT-PLHLEKKLTRAKFEELIQPLLKRLRGPVEQALKDAGLTPAQIDEVILVGGATRVPAVQQVVRELL-GKEPNRSVNPDEVVAM
Vibrio_cholerae                   QTDVNLPYITADA--TG-PKHMNIKVTRAKLEALVEDLVQRSLEPLKVALADADLSVNDITDVILVGGQTRMPMVQKKVAEFF-GKEPRKDVNPDEAVAV
Xanthomonas_campestris            QTEVNLPYVTADA--SG-PKHLNIKLTRAKLEALVEDLVKKSIEPCRTALNDAGLRASDINEVILVGGQTRMPKVQQAVADFF-GKEPRKDVNPDEAVAV
Yersinia_pestis                   QTDVNLPYITADG--SG-PKHMNIKVTRAKLESLVEDLVNRSIEPLKVALQDAGLSVSDIQDVILVGGQTRMPMVQKKVADFF-GKEPRKDVNPDEAVAI
                                     :.  ::            .   ..*: :: :   :.          :  :      :  ::: **  * * :   :      :   . :**** *:
```

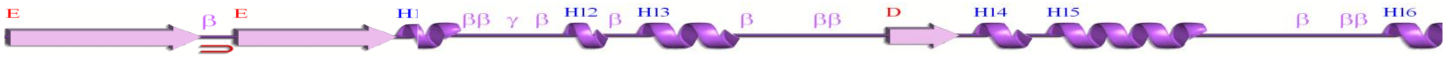

```
Acinetobacter_baumannii         GAAIQGAVLSGD----KNDVLLLDVTPLTLGIETMGGVLTPIIEKNTTIPAKKSQVFSTAADNQPAVDISVYQGERKMAQQNKLLGNFQLGDIPPAPRGV
Agrobacterium_tumefaciens       GAAIQAGVLQGD----VKDVLLLDVTPLSLGIETLGGVFTRLIDRNTTIPTKKSQTFSTAEDNQSAVTIRVSQGEREMAQDNKLLGQFDLVGLPPSPRGV
Aquifex_aeolicus                GAAIQAGVLAGE----VKEIVLVDVTPLSLGVETYGGVMTVLIPRNTPIPVRKCEIFTTAHDYQTEVEIHVLQGERPLAKDNKSLAKFYLTGIPPAPRGV
Bacillus_anthracis              GAAVQGGVLTGD----VEGVLLLDVTPLSLGIETMGGVFTKLIERNTTIPTSKSQVFSTAADNQPAVDIHVLQGERPMSADNKTLGRFQLTDLPPAPRGI
Bacillus_cereus                 GAAVQGGVLTGD----VEGVLLLDVTPLSLGIETMGGVFTKLIERNTTIPTSKSQVFSTAADNQPAVDIHVLQGERPMSADNKTLGRFQLTDLPPAPRGI
Bacillus_subtilis               GAAIQGGVITGD----VKDVVLLDVTPLSLGIETMGGVFTKLIDRNTTIPTSKSQVFSTAADNQTAVDIHVLQGERPMSADNKTLGRFQLTDIPPAPRGV
Bacteroides_thetaiotaomicron    GAAVQGAVLTDE----IKGVVLLDVTPLSMGIETLGGVMTKLIDANTTIPARKSETFSTAADNQSEVTIHVLQGERPMAAQNKSIGQFNLSGIAPARRGV
Bifidobacterium_longum          GAAVQSGVIKGD----RKDVLLIDVTPLSLGIETKGGIMTKLIDRNTAIPTKRSEVFSTAEDNQPSVLIQVYQGEREFARDNKPLGTFELTGIAPAPRGV
Bordetella_pertussis            GAAIQGSVLSGE----RKDVLLLDVTPLSLGIETLGGVMTKMIQKNTTIPTRYSQTFSTADDNQPAVTIKVFQGEREIAAGNKGLGEFNLEGIPPAPRGL
Borrelia_burgdorferi            GAAIQGGILTGE----TKDMVLLDVTPLSLGIETLGGVMTKLIERNTTIPTKKSQVFSTAADNQTSVDIKVLQGEREMAAQNRILGNFILDGIPAAPRGV
Bradyrhizobium_japonicum        GAAIQAGVLQGD----VKDVLLLDVTPLSLGIETLGGVFTRIIDRNTTIPTKKSQVFSTAEDNQNAVTIRVFQGEREMAADNKMLGQFDLMGIPPAPRGM
Buchnera_aphidicola             GAAVQGGVLSGD----VKDVLLLDVTPLSLGIETMGGIMTSLINKNTTIPTKHSQIFSTAEDNQSAVTIHVLQGERKRSSDNKSLGQFNLDGINPAPRGT
Burkholderia_pseudomallei       GAAIQGQVLSGD----RKDVLLLDVTPLSLGIETLGGVMTKMINKNTTIPTKHAQVYSTADDNQGAVTIKVFQGEREMAAGNKLLGEFNLEGIPPAPRGV
Campylobacter_jejuni            GAAIQGAVIKGD----VKDVLLLDVTPLSLGIETLGGVMTKIIEKGTTIPTKKEQVFSTAEDNQSAVTINVLQGEREFSRDNKSLGNFNLEGIPPAPRGM
Caulobacter_crescentus          GAAVQAGVLQGD----VKDVLLLDVTPLTLGIETLGGVFTPLIERNTTIPTKRSQTFSTADDNQSAVTIRVFQGERPMAQDNKMLGQFDLVGIPPAPRGV
Chlamydia_trachomatis           GAAIQGGVLGGE----VKDVLLLDVIPLSLGIETLGGVMTPLVERNTTIPTQKKQIFSTAADNQPAVTIVVLQGERPMAKDNKEIGRFDLTDIPPAPRGH
Chlorobium_tepidum              GAAIQGGVLQGD----VTDVLLLDVTPLSLGIETLGGVMTKLIEANTTIPTRKQEIFSTAADNQTSVEVHVLQGERPMASDNKTLGRFHLSDIPPAPRGV
Clostridium_acetobutylicum      GAAIQAGVLTGD----VKDVLLLDVSPLTLGIETLGGVATPLIERNTTIPTRKSQVFSTAADNQPSVEINIVQGERKMAADNKSLGRFTLDGIAPAPRGV
Clostridium_botulinum           GAAIQAGVLTGE----VKDVLLLDVTPLTLGIETFGGVSTTLIEKNTTIPTRKSQVFSTAADGQTSVEIHVVQGERSMAADNKTLGRFTLSGIAPAPRGI
Corynebacterium_glutamicum      GAALQAGVLRGE----VKDVLLLDVTPLSLGIETKGGVMTKLIERNTTIPTKRSETFTTAEDNQPSVQIQVFQGEREIATANKLLGSFELGGIAPAPRGV
Coxiella_burnetii               GAAIQGAVLSGE----VKDVLLLDVTPLSLGIETLGGVMTKLIEKNTTIPTKANQVFSTADDNQTAVTVHVLQGEREMASANKSLGRFDLSDIPPAPRGV
Deinococcus_radiodurans         GAAVQAGIIQGD--SSLGDIVLVDVTPLTLGVEVKGGMIAPMITRNTTVPAKKTEIYTTAENNQPGVEINVLQGERPMAADNKSLGRFKLEGIPPMPAGR
Desulfovibrio_vulgaris          GAAIQGGILAGD----VKDVLLLDVTPLSLGIETLGGVFTRLIERNTTIPTRKSQTFTTAADNQPSVSIHVLQGERPMASDNMTLGRFELTGIPPAMRGV
Dictyoglomus_turgidum           GAAIQAGVIGGE----IRDIVLVDVTPLSLGIETLGGVFTKIIERNTPIPVSKSQIFTTAADYQTSVEIHVLQGERALAKDNISLGRFILDGIPPAPRGV
Enterococcus_faecalis           GAAIQGGVITGD----VKDVVLLDVTPLSLGIETMGGVFTKLIDRNTTIPTSKSQVFSTAADNQPAVDIHVLQGERPMAADNKTLGRFQLTDIPAAPRGV
Escherichia_coli                GAAVQGGVLTGD----VKDVLLLDVTPLSLGIETMGGVMTTLIAKNTTIPTKHSQVFSTAEDNQSAVTIHVLQGERKRAADNKSLGQFNLDGINPAPRGM
Flavobacterium_psychrophilum    GAAIQGGVLSGD----VKDVLLLDVTPLSLGIETMGGVMTILIESNTTIPTKKSQIFSTAADSQPTVELHVLQGARAMAVDNKTIGRFNLDGIPPAPRGV
Francisella_tularensis          GAAIQGGVLAGD----VKDILLLDVTPLSLGIETMGGVMTKLIERNTTIPTKKSQVFSTAEDNQPAVTIHVLQGEREMASANKSLGRFDLADIPPAPRGM
Fusobacterium_nucleatum         GAAIQGGVLMGD----VKDVLLLDVTPLSLGIETLGGVFTKMIEKNTTIPVKKSQVYSTAVDNQPAVTINVLQGERSRATDNHKLGEFNLEGIPAAPRGV
Geobacter_sulfurreducens        GAAIQGGVLRGD----VKDVLLLDVTPLSLGIETLGSVMTKLIEKNTTIPCRKSQVFSTASDNQPAVTIHVLQGEREMAIDNKTLGNFELTGIPPAPRGV
Gloeobacter_violaceus           GAAIQAGVLSGE----VRDVVLLDVTPLSLGVETLGGVATPIIPRNTTIPTRKSETFSTAADGQTSVEIHVIQGERSMAGDNKSLGRFRLDGIPPAPRGV
Haemophilus_influenzae          GAAVQGGVLKGD----VKDVLLLDVTPLSLGIETMGGVMTTLIEKNTTIPTKKSQVFSTAEDNQSAVTIHVLQGERKRAADNKSLGQFNLEGINPAPRGM
Helicobacter_pylori             GASIQGGVLKGD----VKDVLLLDVTPLSLGIETLGGVMTKVIDRGTTIPAKKSQVFSTAEDNQPAVSIMVLQGERELARDNKSLGKFDLQGIAPAPRGV
Koribacter_versatilis           GAAIQGGVLKGE----VKDLLLLDVTPLTLSIETLGGVATPMIPRNTTIPTKKTETFSTAADNQNSVEIHVLQGERPMAGQNRTLGKFHLTGLPPAPRGV
Lactobacillus_plantarum         GAAVQGGVITGD----VKDVVLLDVTPLSLGIETMGGVFTKLIDRNTTIPTSKSQVFSTAADNQPAVDIHVLQGERPMAADNKTLGRFQLTDIPAAPRGV
Lactococcus_lactis              GAAIQGGVITGD----VKDVVLLDVTPLSLGIETMGGVFTKLIDRNTTIPTSKSQVFSTAADNQPAVDIHVLQGERPMAADNKTLGRFQLTDIPAAPRGI
Leptospira_interrogans          GAAIQGGVLAGE----VSDVLLLDVTPLSLGIETLGGVMTKLIERNTTIPTKKSQVFSTAADNQSAVSIHVLQGEREMASANRTLGRFDLIGIPPAPRGV
Listeria_monocytogenes          GAAIQGGVITGD----VKDVVLLDVTPLSLGIETMGGVMTTLIERNTTIPTSKSQTFSTAADNQPAVDIHVLQGERPMAKDNKTLGRFQLADIPPAPRGI
Mesoplasma_florum               GAAIQGGVLAGD----VTDVLLLDVTPLSLGIETMGGVMTKLIDRNTTIPTEKSQIFSTAVDNQPAVDINVLQGERPMAADNKSLGQFQLTGIKPAPKGT
Moorella_thermoacetica          GAAIQGGVLAGE----VKDVLLLDVTPLSLGIETLGGVFTKLIERNTTIPTSKSQIFSTAADNQTTVEIHVLQGERAMAADNKTLGRFQLTGIPPAPRGV
Mycobacterium_smegmatis         GAALQAGVLKGE----VKDVLLLDVTPLSLGIETKGGVMTKLIERNTTIPTKRSETFTTADDNQPSVQIQVYQGEREIASHNKLLGSFELTGIPPAPRGV
Mycobacterium_tuberculosis      GAALQAGVLKGE----VKDVLLLDVTPLSLGIETKGGVMTRLIERNTTIPTKRSETFTTADDNQPSVQIQVYQGEREIAAHNKLLGSFELTGIPPAPRGI
Mycoplasma_mycoides             GAAVQGGVLAGE----VTDILLLDVTPLSLGIETMGGVMTKLIERNTTIPAKRTQIFSTATDNQPAVDINVLQGERAMAADNKSLGQFQLTGIQPAPRGV
Mycoplasma_pneumoniae           GAAIQGGVLRGD----VKDVLLLDVTPLTLSIETLGGVATPLIKRNTTIPVSKSQIFSTAQDNQESVDVVVCQGERPMARDNKSLGRFNLGGIQPAPKGK
Neisseria_gonorrhoeae           GAAIQGEVLSGG----RSDVLLLDVTPLSLGIETMGGVMTKLIQKNTTIPTKASQVFSTAEDNQSAVTIHVLQGERERASANKSLGQFNLGDIAPAPRGM
Neisseria_meningitidis          GAAIQGEVLSGG----RSDVLLLDVTPLSLGIETMGGVMTKLIQKNTTIPTKASQVFSTAEDNQSAVTIHVLQGERERASANKSLGQFNLGDIAPAPRGM
Pseudomonas_aeruginosa          GAAIQGAVLAGD----VKDVLLLDVTPLTLGIETLGGVMTGLIEKNTTIPTKKSQVFSTADDNQGAVTIHVLQGERKQAAQNKSLGKFDLADIPPAPRGV
Rhodobacter_sphaeroides         GAAIQAGVLQGD----VKDVVLLDVTPLSLGIETLGGVFTRLIDRNTTIPTKKSQVFSTAEDNQNAVTIRVFQGEREMAADNKLLGQFNLEDIPPAPRGM
Rhodopirellula_baltica          GAAIQGSVLAGD----RNDVLLLDVTPLTLGIETEGGVMTALVERNTTIPAEKKNVFSTAADNQTAVTVRVFQGERKMANANRLLAEFNLEDIPAAPRGV
Rhodospirillum_rubrum           GAAIQGGVLKGD----VKDVLLLDVTPLSLGIETLGGVFTRLIDRNTTIPTRKSQTFSTAEDSQTAVTIRVFQGEREMAADNKMLGQFDLVGLPSAPRGV
Rickettsia_prowazekii           GAAIQGGVLNKE----VTDILLLDVTPLSLGIETLGGVFTRLIDRNTTIPSKKSQVFSTADDNQHAVTIRVFQGEREMAKDNKLLGQFNLEGIPPAPRGV
Salinibacter_ruber              GAAVQGGVLSGD----VDDVLLLDVTPLNLGIETLGGVMTTLIEANTTIPTKESEVFSTAADNQTSVEVHVLQGDREMAKDNRTLGRFHLDGIPPAPRGT
Salmonella_typhimurium          GAAVQGGVLTGD----VKDVLLLDVTPLSLGIETMGGVMTPLITKNTTIPTKHSQVFSTAEDNQSAVTIHVLQGERKRASDNKSLGQFNLDGINPAPRGM
Shewanella_oneidensis           GAAIQAGVLSGD----VKDVLLLDVTPLSLGIETMGSVMTKLIEKNTTIPTKAQQVFSTADDNQSAVTIHVLQGERKQASANKSLGQFNLDGIEPAPRGM
Shigella_dysenteriae            GAAVQGGVLTGD----VKDVLLLDVTPLSLGIETMGGVMTTLIAKNTTIPTKHSQVFSTAEDNQSAVTIHVLQGERKRAADNKSLGQFNLDGINPAPRGM
Staphylococcus_aureus           GAAIQGGVITGD----VKDVVLLDVTPLSLGIEILGGRMNTLIERNTTIPTSKSQIYSTAVDNQPSVDVHVLQGERPMAADNKTLGRFQLTDIPPAERGK
Streptococcus_pneumoniae        GAAIQGGVITGD----VKDVVLLDVTPLSLGIETMGGVFTKLIDRNTTIPTSKSQVFSTAADNQPAVDIHVLQGERPMAADNKTLGRFQLTDIPAAPRGI
Streptomyces_coelicolor         GAALQAGVLKGE----VKDVLLLDVTPLSLGIETKGGIMTKLIERNTTIPTKRSEIFTTAEDNQPSVQIQVYQGEREIAAYNKKLGMFELTGLPPAPRGV
Synechocystis_sp.               GAAIQGGVLSGE----VKDILLLDVSPLSLGVETLGGVMTKIIPRNTTIPTKKSETFSTAVDGQSNVEIHVLQGEREMANDNKSLGTFRLDGIPPAPRGV
Thermodesulfovibrio_yellowstonii GAAIQAAILKGE----VKEVLLLDVTPLSLGIETLGGVFTKIIERNTTIPTKKSQIFTTAADNQTAVTIKVYQGEREMAADNKLLGVFELVGIPPAPRGI
Thermotoga_maritima             GAAIQAAILAGTEGAKGRDIVLVDVTPLTLGIEVKGGLFEPIIPRNTKIPVRKSKIFTTVEDGQTEVEIRVYQGERPIARENIFLGSFKLVGIPPAPRGV
Thermus_thermophilus            GAAIQAGVLMGE----VRDVVLLDVTPLSLGVETKGGVMTVLIPRNTTIPTRKCEIFTTAEHNQTAVEIHVLQGERPMAQDNKSLGRFRLEGIPPMPAGV
Vibrio_cholerae                 GAAVQGGVLAGE----VKDVLLLDVTPLSLGIETMGGVMTKLIEKNTTIPTKANQVFSTAEDNQSAVTIHVLQGERKQAMYNKSLGQFNLEGINPAPRGM
Xanthomonas_campestris          GAAIQGGVLAGD----VKDVLLLDVTPLSLGIETMGGVFTKIIEKNTTIPTKASQTFSTAEDNQSAVTVHVLQGEREQARFNKSLAKFDLSGIEPAPRGM
Yersinia_pestis                 GAAVQGGVLSGE----VKDVLLLDVTPLSLGIETMGGVMTPLITKNTTIPTKHSQVFSTAEDNQSAVTIHVLQGERKRAQDNKSLGQFNLDGIQPAPRGM
                                **::*. ::          ::*:** **.:.:*  *.    ::   * :*    : ::*. . *  * : : ** *  :  *  :. * *  :     *
```

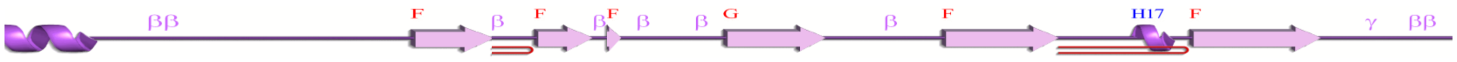

```
Acinetobacter_baumannii            PQIEVSFDINADGILKVSAKDKSTGKEQSIQIKANSGLSDAEIEAMIKDAEANAEEDRKFEELAKARNEADALISSSNKAVKDLGDK---------VTEDE
Agrobacterium_tumefaciens          PQIEVTFDIDANGIVQVSAKDKGTGKEQQIRIQASGGLSDADIEKMVKDAEANAEADKKRRAGVEAKNQAESLIHSTEKSVKEYGDK---------VSETD
Aquifex_aeolicus                   PKIEVCFDIDADGILHVTAKDLGTGKEQSVRVEISSGLTPEEIERIIKEAEEHAEEDRKKKELIEAKNQLDHLVYQLEKALKEAGDK---------VPADV
Bacillus_anthracis                 PQIEVTFDIDANGIVNVRAKDLGTSKEQAITIQSSSGLSDEEVERMVQEAEANADADQKRKEEVELRNEADQLVFQTDKVVKDLEGK---------VDAAE
Bacillus_cereus                    PQIEVTFDIDANGIVNVRAKDLGTSKEQAITIQSSSGLSDEEVERMVQEAEANADADQKRKEEVELRNEADQLVFQTDKVVKDLEGK---------VDAAE
Bacillus_subtilis                  PQIEVSFDIDKNGIVNVRAKDLGTGKEQNITIKSSSGLSDEEIERMVKEAEENADADAKKKEEIEVRNEADQLVFQTEKTLKDLEGK---------VDEEQ
Bacteroides_thetaiotaomicron       PQIEVTFDIDANGILKVSAKDKATGKEQAIRIEASSGLSKEEIEKMKAEAEANAEADKKEREKIDKLNQADSVIFQTENQLKELGDK---------LPADK
Bifidobacterium_longum             PQIEVTFDIDANGIVHVSAKDKGTGKEQSMTITGGSGLPKDEIDRMVKEAEAHEAEDKKRKEDAETRNQAEAFAYSTEKLVNDNKDK---------LSDDI
Bordetella_pertussis               PQIEVTFDIDANGILHVSAKDKGTGKENKITIKANSGLSEDEIQRMVKDAEANAEEDHRLAELAQARNQADALVHATRKSLTEYGEK---------LEAAE
Borrelia_burgdorferi               PQIEVSFDIDANGIVHVSAKDMGTGKEQKIRIESSSGLSESEIDRMVKDAEAHAEEDKKLKENIEAKNTANSLIYQTEKSLKEYSEK---------ISSED
Bradyrhizobium_japonicum           PQIEVTFDIDANGIVNVSAKDKATGKEQQIRIQASGGLSEADIEKMVKDAEANAEADKKRREAVTAKNEADGLVHSTEKALAEHGSK---------VAETE
Buchnera_aphidicola                AQIEVTFDIDSDGILHVSAKDKKTGKEQKITIKASSGLNEEEIKKMVNDAEANSEADQKFEELIQTRNQGDQLVHSIKKQLNENKNS---------IEEES
Burkholderia_pseudomallei          PQIEVTFDIDANGILHVGAKDKATGKENKITIKANSGLSEAEIEKMVKDAEANAAEDHKLRELAESRNQGDALVHSTKKALTEYGDK---------LEAGE
Campylobacter_jejuni               PQIEVTFDIDANGILTVSAKDKATGKAQEIKITGSSGLSEEEINNMVKDAELHKEEDKKRKEAVDARNAADSLAHQVEKSLSELGEK---------VAAAD
Caulobacter_crescentus             PQIEVTFDIDANGIVQVHAKDKATNKEQSIRIQANGGLSDSDIERMVKEAEANKAADEKKKQLVEAKNQGEAILHSTEKALAEHGDK---------VGAAE
Chlamydia_trachomatis              PQIEVTFDIDANGILHVSAKDAASGREQKIRIEASSGLKEDEIQQMIRDAELHKEEDKQRKEASDVKNEADGMIFRAEKAVKDYHDK---------IPAEL
Chlorobium_tepidum                 PQIEVTFDIDANGILNVSAKDKATGKEQSIKIEASGKLTEAEIEKMKEDAKAHAAEDQKRKEEIELKNSADSLIFSTEKQLTELGDK---------LPADK
Clostridium_acetobutylicum         PQIEVTFDIDANGIVNVSAKDKGTGKESHITITASTNLSDEEIDKAVKDAEKFAEEDKKKKENIEVKNNADQIVFQTDKALKDLGDK---------VSAED
Clostridium_botulinum              PQIEVTFDIDANGIVNVSAKDKGTGKEANITITASTNLTDDEIEKAVNEAKKFEAEDKKRKESIEIKNNADQIVYQTEKTLTDLGDK---------VSAED
Corynebacterium_glutamicum         PQIEVTFDIDANGIVHVTAKDKGTGKENTITIQDGSGLSQDEIDRMIKDAEAHADEDKKRREEQEVRNNAESLVYQTRKFVEENSEK---------VSEDL
Coxiella_burnetii                  PQIEVTFDIDANGILHVSAKDKATGKEQSIVIKASSGLSDEEVEKMVKDAEAHRDSDRKFHELVDARNQADAMIHAAEKSVKDLGSE---------VSADE
Deinococcus_radiodurans            AQIEVTFDIDANGILHVTAKEKTSGKESSITIENTTTLDKTDVERMVQEAEQNAAADKQRKEKVEKRNNLDSLRVQAVQQLEEQEGA----------AQDA
Desulfovibrio_vulgaris             PQIEVSFDIDANGIVNVAAKDLGTGKEQSIRITASSGLSEDEIQRLVKEAEAHADDDKKKQELIEARNQADGLIYGTEKSIRDLGDK---------LDAAL
Dictyoglomus_turgidum              PQIEVTFDIDVNGIVHVSAKDKATGREQRITISNAIRLSEAEIKRMTEEAKRFEEEDRKRREEIETKNQAEHLIYTARKTLKDYGDK---------VSKDI
Enterococcus_faecalis              PQIEVSFDIDKNGIVNVRAKDLGTQKEQTITIKSSSGLSDEEIERMVKDAEANAEADKQRKEEVDLRNDADALLFTVDKTLKELEGK---------VDAEE
Escherichia_coli                   PQIEVTFDIDADGILHVSAKDKNSGKEQKITIKASSGLNEDEIQKMVRDAEANAEADRKFEELVQTRNQGDHLLHSTRKQVEEAGDK---------LPADD
Flavobacterium_psychrophilum       PQIEVAFDIDANGIIKVSATDKGTGKSHDIRIEASSGLTSEEIERMKKDAEANAGADKIARERVEKINEADSLIFQTETQLKELGDK---------ITDEH
Francisella_tularensis             PQIEVTFDIDANGILNVSAKDKATGKEQNIVIKSSSGLSEEDIEKMVQDAEANAEADKKFHDLVTARNTADNLIHSSRKAIQELGDK---------VTAAE
Fusobacterium_nucleatum            PQIEVTFDIDANGIVHVSAKDLGTGKENKVTISGSSNLSKEEIERMTKEAEAHAEEDKKFQELVEARNKADQLISATEKTLKENPDK---------VSEED
Geobacter_sulfurreducens           PQIEVTFDIDANGIVHVSAKDLGTGKEQSIRITASSGLSKEEIDKMVKEAEAHSAEDKKKRELVEARNHADTLSYSTEKSLKEYGDK---------IGADE
Gloeobacter_violaceus              PQVEVTFDIDANGILSVTAKDKASGKAQTISITGASTLSKDDVAKMVNEAESFAGEDKKRREAVDLKNEADSLAYQAERQLTEFGDK---------VDSSD
Haemophilus_influenzae             PQIEVTFDIDANGVINVSAKDKNTGKEQQIRIQASSGLSDEEIQQMVRDAEANADADRKFEEVVQARNQADGIAHATRKQIAEAGDA---------LSVAD
Helicobacter_pylori                PQIEVTFDIDANGILTVSAQDKNTGKSQEIKISGSSGLSDSEIEKMVKDAELHKEEDAKKKEVIEARNHADSLAHQTQKSLDEHKTN---------LNEND
Koribacter_versatilis              PQIEVTFDIDANGILNVTAKDNATGKDQKITITSSSGLSKEEVERMAKEADAHAEEDKAKKEEIEARNQLDGMVYQIEKMLKENGDK---------ISPSE
Lactobacillus_plantarum            PQIEVKFDIDKNGIVNVSAKDMGTNKEQKITIKSSSGLSDDEIDQMVKEAKENEEADKKRKEEVDLKNEVDQLIFTTDKTLKDLEGK---------VSEDE
Lactococcus_lactis                 PQIEVTFDIDKNGIVSVKAKDLGTQKEQTIVIKSNSGLSDEEIDKMMKDAEANADADAKRKEEVDTRNEADALVFQTEKTLKDLEGK---------VEEAE
Leptospira_interrogans             PQIEVTFDIDANGIVHVSAKDLGTGKEQKIRIESSSGLSEDEIQKMVKDAEAHAAADKAQREVIEAKNELDTLTYSLEKTVNEAGDK---------IGANE
Listeria_monocytogenes             PQIEVSFDIDKNGIVTVRAKDLGTGKEQNIVIKSSSGLTDEEIEKMVQDAEANAEEDKKNKENAELRNNADQLVFTVDKTLKELEGK---------VEEEE
Mesoplasma_florum                  PQIEVTFKIDVNGIVSVIAKDKATNEEKSITISNSGALSDADIEKMIKEAEANAEADEIKRKNIELKHKAESYVAIIESSLTQEGQE---------IPQEQ
Moorella_thermoacetica             PQIEVKFDIDANGIVHVSAKDLGTGKQQAITITSSSGLSEEEIQRMVKEAEASAEADRRRKEEIETRNQADSLIYQAERTLKEFKDK---------ADQND
Mycobacterium_smegmatis            PQIEVTFDIDANGIVHVTAKDKGTGKENTIKIQEGSGLSKEEIDRMIKDAEAHAEEDRKRREEADVRNQAESLVYQTEKFVAEQRGAASDGGGSKVPEET
Mycobacterium_tuberculosis         PQIEVTFDIDANGIVHVTAKDKGTGKENTIRIQEGSGLSKEDIDRMIKDAEAHAEEDRKRREEADVRNQAETLVYQTEKFVKEQREA---EGGSKVPEDT
Mycoplasma_mycoides                PQIEVTFEIDANGIVSVSAKDKNTNEEKTITISNSGNLSEAEVERMIKEAQENAANDEVKKKNIELKNKAENYINIIENSLLQAGDK---------ISAEQ
Mycoplasma_pneumoniae              PQIEITFSLDANGILNVKAKDLTTQKENSITISDNGNLSEEEIQKMIRDAEANKERDNVIRERIELRNEGESIVSTIKEILQSPEAK--------DFPKEE
Neisseria_gonorrhoeae              PQIEVTFDIDANGILHVSAKDKGTGKAANITIQGSSGLSEEEIERMVKDAEANAEEDKKLTELVASRNQAEALIHSVKKSLADYGDK---------LDAAE
Neisseria_meningitidis             PQIEVTFDIDANGILHVSAKDKGTGKAANITIQGSSGLSEEEIERMVKDAEANAEEDKKLTELVASRNQAEALIHSVKKSLADYGDK---------LDAAE
Pseudomonas_aeruginosa             PQIEVTFDIDANGILHVSAKDKATGKQQSIVIKASSGLSEDEIQQMVRDAEANAEEDRKFEELAAARNQGDALVHATRKMITEAGDK---------ATAED
Rhodobacter_sphaeroides            PQIEVTFDIDANGIVSVSAKDKGTGKSQNITIQASGGLSDEDIEKMVRDAEANAEADKKRRELVETKNQGESLLHSTRKSIEEHGDK---------VDPST
Rhodopirellula_baltica             PQIEVKFDIDQNGILSVSAKELKTGKEANVEIKDSGALSDSDIEQMQKDAEANAEEDKRQFELVEARNKVNQQVYQLEKLMGENDDK---------LSDDD
Rhodospirillum_rubrum              PQIEVTFDIDANGIVNVSAKDKATGKEQAIRIQASGGLSDNDIERMVKEAELNAEADRKRKEAVEARNHADGLIHATEKNLKEYGDK---------IPAED
Rickettsia_prowazekii              PQIEVTFDIDANGIVHVSAKDKASGKEQKVTIQASGGLSDAEIEQMVKDAEHNADEDKKRKELIETKNAADSLVYSTEKTLREYGDK---------LSSEE
Salinibacter_ruber                 PQIEVTFDINADGILNVSAEDKDTGKEQSIRVEANSGLSDEEIEKMKEEAEQHAEEDERRKERADTINEANSMAYSVEQGLEEYGDK---------IPEDK
Salmonella_typhimurium             PQIEVTFDIDADGILHVSAKDKNSGKEQKITIKASSGLNEEEIQKMVRDAEANAESDRKFEELVQTRNQGDHLLHSTRKQVEEAGDK---------LPADD
Shewanella_oneidensis              PQIEVMFDIDADGILHVSATDKKTGKKQNITIKASSGLSEEEVAQMVRDAEANAEEDKKFEELVQSRNQADGLVHATKKQVEEAGDA---------LPSED
Shigella_dysenteriae               PQIEVTFDIDADGILHVSAKDKNSGKEQKITIKASSGLNEDEIQKMVRDAEANAEADRKFEELVQTRNQGDHLLHSTRKQVEEAGDK---------LPADD
Staphylococcus_aureus              PQIEVTFDIDKNGIVNVTAKDLGTNKEQRITIQSSSSLSDEEIDRMVKDAEVNAEADKKRREEVDLRNEADSLVFQVEKTLTDLGEN---------IGEED
Streptococcus_pneumoniae           PQIEVTFDIDKNGIVSVKAKDLGTQKEQTIVIQSNSGLTDEEIDRMMKDAEANAEADKKRKEEVDLRNEVDQAIFATEKTIKETEGK---------GFDAE
Streptomyces_coelicolor            PQIEVAFDIDANGIMHVTAKDLGTGKEQKMTVTGGSSLPKDEVDRMRQEAEKYAEEDHARREAAESRNQGEQLVYQTEKFLKDNEDK---------VPGEV
Synechocystis_sp.                  PQIEVTFDIDANGILNVTAKDRGTGKEQSISITGASTLPDTEVDRMVKEAESNAAADKERREKIDRKNQADSLVYQAEKQITELGDK---------VPAAD
Thermodesulfovibrio_yellowstonii   PQIEVTFDIDANGILHVSAKDLATGKEQSIRITASSGLSEEEIKKMIREAEAHAEEDRRKKQIAEARNEADNMIYTVEKTLRDMGDR---------ISEDE
Thermotoga_maritima                PQIEVTFDIDSDGIVHVSAKDLGSGKEQSMVVTGRHKLSEDEIKRMIEDAKRYEEQDKRLKEEIELKNRADDLAYSVEKTLKEHGDK---------IPADL
Thermus_thermophilus               PQIEVCFDIDANGILHVTAKERSTGREASITIQNTTTLSEEEIQRIIEEAKRHAEEDRRRREHAELKNALDSARVQAERVLQERQGA----------PE-A
Vibrio_cholerae                    PQIEVIFDLDADGILHVSAKDKQTGKEQKITIQASGGLSDAEIEKMVQEAEANKEADKKFEELATARNQADQMIHATRKQITEAGEA---------LPADE
Xanthomonas_campestris             PQVEVSFDIDANGILHVSAKDKKTNKEQKVEIKAGSGLSDEEIQRMVADAEANREEDKKFQELVQTRNQADGLIHATRTAITEHGSK---------VGGDV
Yersinia_pestis                    AQIEVTFDIDADGILHVSAKDKNTGREQKITIKASSGLNEEEIQKMVRDAEANAEADRKFEELVQTRNQADHLIHGTRKQLEEAGDK---------LPAED
                                    ::*: *.:: :*:: * * :  : .   : :     *   ::     :*.     *          .  :          : .
```

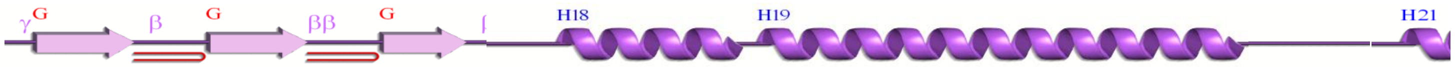

```
Acinetobacter_baumannii          KTAVNTAVSELEAATKE----N--DVEAIKAKTEALQNILMPITQRAYEQAQQAGGAEGF----DPN-------------------AFQGGDAGQQKADD
Agrobacterium_tumefaciens        RKAIEDAIASLKTAVEA--AEP--DADDIQAKTQTLMEVSMKLGQAIYEAQQAEAGDASA------------------------------------EGKD
Aquifex_aeolicus                 KSEAEKVIEEAKKTIETAT-----EIEQVKQVTEKVLQVS-SKMGT----TLYGEA--GK-------------------------------QAGGGEKKD
Bacillus_anthracis               VAKATEAKEALQAAIEK----N--ELEEIRAKKDALQEIVQQLTVKLYEQAQAAAGQAEG-------------AEGA---------------QDAGAKKD
Bacillus_cereus                  VAKATEAKEALQAAIEK----N--ELEEIRAKKDALQEIVQQLTVKLYEQAQAAAGQAEG-------------AQGA---------------QDAGAKKD
Bacillus_subtilis                VKKANDAKDALKAAIEK----N--EFEEIKAKKDELQTIVQELSMKLYEEAAKAQQA----------------QGGA---------------NAEGKADD
Bacteroides_thetaiotaomicron     KAPIEAALQKLK----DAHKSQ--DLAAIDTAMAEINTVFQTASAEMYAQGGAQGG--AQA--------GPDMDAGQNAGQD------------NSKHGD
Bifidobacterium_longum           VKEVTDKVNALKEALKG----D--DTEKVKTAQTELMTAAQKIGQVLYAQQGAEGAAAGAD---------------G---------AGASA-GSASGSDD
Bordetella_pertussis             KESIEAAIKDLEDILKT----G--DKAEIDAKVEALSTASQKLGEKMYADMQAQQQAQQQQA-----------------------------ADNAKPVDD
Borrelia_burgdorferi             KEAIESKIKELKESLEK----E--DISLIKSRTEELQKASYKIAEMMYKDSSQQNANSQQEN--G----------------------------PQSNTSE
Bradyrhizobium_japonicum         RRAIEDAVSDLKEALKG----D--DAEAIKAKTQTLAQASMKLGEAMYKQQA-EADAKKD------------------------------------AAKD
Buchnera_aphidicola              KKDIQLALNKLENALKG----E--DKSDIEKNIQNLLKISSKLTEINQKKSEKDQK---------------------------------DNNMSANKKDE
Burkholderia_pseudomallei        KEKIEAALKELEDVLKN--ASS--DKAAIDAKVEAVATASQKLGEKMYADMQAQQAGAAGAA--GAA-------------------AEGASAQGGAQPAD
Campylobacter_jejuni             KENIQKALDDLRETLKN--QNA--SKEEIESKMKALSEVSHKLAENMYKKDEPNTA------------------------------------NDKKKKDD
Caulobacter_crescentus           KTAIETGITELKTALEG----E--DVEAIQAKTQALIQASMKLGEAMYAAQQGSAEGGD------------------------------------AKADD
Chlamydia_trachomatis            VKEIEEHIEKVRQAIKE--D-A--STTAIKAASDELSTHMQKIGEAMQAQSASAAASSAANAQGGPNINSEDLK--------KHSFSTRPPAGGSASSTD
Chlorobium_tepidum               KAAIESALEKLK----EAHKSG--RVDAIKPAMDELSKVWSDAASNLYGQPGAEPQ--PET--------N--------GHAG------------GSKGGD
Clostridium_acetobutylicum       KSNIEAKKEALSKVKDG----D--DIEAIKKATEDLTQALYAITTKMYEQSGAQGAP-GADP------------------------NAGASQKTNGGADD
Clostridium_botulinum            KAQIEEKVKAVKDVKDG----E--DLEAIKKATEDLTQTFYGISSKIYQQANPEGAQ-GAGFD-PNN------MGGA---------NAGNASAGNDKKDD
Corynebacterium_glutamicum       KAKVEEAAKGVEEALKG----E--DLEAIKAAVEKLNTESQEMGKAIYEADAAAGATQA-------------------------------DAGAEGAADD
Coxiella_burnetii                KSAIEKAVNELKEAMKG----N--DKDAIEAKTKALTEHSSKLAERVYAKKGGAAGAPPG----GE--------------------AEGEPQAQAGGKKE
Deinococcus_radiodurans          KDRLKAAADEAEEAVRSE------DDSKIADAQKKLEEELRSFMTANQASTQGQPE--GT-------------------------------QAQANKADD
Desulfovibrio_vulgaris           KADIETKVTALRGLLES----E--DVDAIKKASDELAQASHKLAEQLYKQQAQAGGPEAGAQ--PEG------------------------DAGARKQDD
Dictyoglomus_turgidum            VQKVEDKIKNLEELIKP----ERINVEQVRKGMEELTQTLGEIGQFMYQSAGSTAGNPGQGQS-TEN------------------------------PGG
Enterococcus_faecalis            VKKAEDARDELKAAIEA----N--DIEQMKAKRDSLNEIVQNLTVKLYEQAAQQQAQENP--------------------------------EAAQGGAD
Escherichia_coli                 KTAIESALTALETALKG----E--DKAAIEAKMQELAQVSQKLMEIAQQQHAQQQT------------------------------AG-ADASANNAKDD
Flavobacterium_psychrophilum     KTAIEYALTELR----MAHQSQ--DLEAIQKGLDNVNAAWKTATEAMYAQGEQGQA--A--------------------QPQ------------AETQGD
Francisella_tularensis           KEKIEEACKELEAATKG----D--DKQAIESKTKALEEAFAPIAQKAYAEQAQAAVAQ------------------------------GGAKAEEPKKEE
Fusobacterium_nucleatum          KKNIEAAIEELKKVKDG----D--DKSAIDSAMEKLSQASHKFAEELYKEVQAQAQA-QQ-------------QAGA---------NAGSDKKDEDVAEA
Geobacter_sulfurreducens         KAKIEECLANLRKAMEG----S--DVEVLKKATDELTQASHKLAEAVYAKAQAEGAQPGG-----EA------------------------AGEASAKDE
Gloeobacter_violaceus            KSKIEGLIKDLREALSR----E--DMDKVASLKADLQQAVYDLSSKLYQQSAPSGAAAGP-------------DEGA---------PSGSGGTSGTRGGD
Haemophilus_influenzae           KEKIEAAVAELETAAKG----E--DKAEIEAKIEAVIKASEPLMQAVQAKAQQAGG---------------------------------EQPQQSSAKDD
Helicobacter_pylori              ANEIQNAINALKDCVKN--DNA--TKAELEDKTKLLAQAAQKLGEAMANKN---NA------------------------------------EQPKKKDD
Koribacter_versatilis            RGDVENALADSKKALET----N--DAKQMNAARERLTAASHKLAEAMYKQAAPGAGAPGAGP--GPEAA-----------------GGAQQAQAEPKKDE
Lactobacillus_plantarum          VKKAKDARDALKKAQDD----N--NIDEMKAKKDDLNKIVQDLSVKLYQQAQEAQGAQGG-------------ADSN---------AAGN-ANSAKGSDD
Lactococcus_lactis               VKKAEEAKEELKKALEG----E--DIDDIKAKSEALSEIAQNLAVKLYEQANAAQGEAGQ-------------ATDA---------------Q-EAPKDD
Leptospira_interrogans           KQLATDEIKRAREAIES----N--DKARIESAKASISKIATDIASKVYSQSAPGAEQAAAGG--PNG-S-----------------AGSNDQGNSTNNGE
Listeria_monocytogenes           VKKAEAARDELQEALKG----E--DFDAIKEKTESLNEIVQNLSVKLYEQAAAEQQAAGG-------------AEGQ---------------E--APQND
Mesoplasma_florum                KEQAEKMVAEVKELIEK----E--DYEALETKVNELEQMIQMASQFAQAQTAQPEEGNSD--S-EEN------K--------------------------
Moorella_thermoacetica           VDRIEKAKKELQEVLDS----K--NNDKIKEKMEALSQALYTLTTKVYQQAGAQAGA------------------------------QGQGAAGGQKQDG
Mycobacterium_smegmatis          LAKVDSAIADAKKALEG----T--DISAIKSAMEKLGVESQALGQAIYEATQAEQPAGGSD-------------------------------NGAPG-DD
Mycobacterium_tuberculosis       LNKVDAAVAEAKAALGG----S--DISAIKSAMEKLGQESQALGQAIYEAAQAASQATGAA---------------H---------PGGEPGGAHPGSAD
Mycoplasma_mycoides              KEQSQKMVDEIKELVKN----E--NYEALEQKMAELEQAMAQAAEFANKQNESDPNNNSS--E-QNN---------------------------------
Mycoplasma_pneumoniae            KEKLDKITGGIDAAIKA----N--DYTKLKAEIENFKKWREEMAKK-YNPNGDQGQPAQ-----------------------------------------
Neisseria_gonorrhoeae            KEKIEAALKEAEEAVKG----D--DKTAIDAKAEALGTASQKLGEMVYAQAQAEAQA-----------------------------GEGAQANASAKKDD
Neisseria_meningitidis           KEKIEAALKEAEEAVKG----D--DKAAIDAKTEALGAASQKLGEMVYAQAQAEAQA-----------------------------GESEQANASAKKDD
Pseudomonas_aeruginosa           KATIEKALGELEAAVKG----D--DKAEIEAKMNALSQASTPLAQKMYAEQAQQGE--------------------------------DAPQGEQAKAAD
Rhodobacter_sphaeroides          VEAIELAMGALEEALKS----E--DAGKIKGGIQNLTEAAMRLGEAIYKASQAEGGATPD-E------------------------------EGPRSVDD
Rhodopirellula_baltica           KAPMNAAIEKVKKAAEG----D--DLAEIKAASDELEAASQAFSKVLYEKTDAAGEAGAD----------------A---------AGAAGATAGGGDDD
Rhodospirillum_rubrum            KAKVEGDLTALKAVLDS----E--DAESIKAKTDALMQSAMKLGEAAYSAGQSAEGAPHA-A--GAE------------------------ASAQSRTDD
Rickettsia_prowazekii            KGTVEEALTSLKAALES----E--DASLIKEKTGNLTAANMKIGEAMYKTQTENQHS-----------------------------------EANTVNNE
Salinibacter_ruber               RSTLQEALDALNEELETASADE--DITALEDALEELNAAWSAAGEEIREAQQQQAQQGAAAGAGA-GAAGAGAAAGAEGPAGGPTGGPASGNGAADSDEE
Salmonella_typhimurium           KTAIESALNALETALKG----E--DKAAIEAKMQELAQVSQKLMEIAQQQHAQQQA------------------------------GS-ADASANNAKDD
Shewanella_oneidensis            KAKIEAAMSAVETAVKG----N--DKEAIEKATQALIEASAKLMEIAQAKATQTGG------------------------------AQEGAAKQSNATAD
Shigella_dysenteriae             KTAIESALTALETALKG----E--DKAAIEAKMQELAQVSQKLMEIAQQQHAQQQT------------------------------AG-ADASANNAKDD
Staphylococcus_aureus            KKSAEEKKDALKTALEG----Q--DIEDIKSKKEELEKVIQELSAKVYEQAAQQQQQAQG-------------A------------------NAGQNNDS
Streptococcus_pneumoniae         RDAAQAALDDLKKAQED----N--NLDDMKAKLEALNEKAQGLAVKLYEQAAAAQQAQEG-------------AEGA---------------QATGNAGD
Streptomyces_coelicolor          KTEVESAVAELKEKLKG----E--DTAEIRTATEKVAAVSQKLGQAMYADAQAAQAAGG-E---------------A---------PGADAGAEGKGADD
Synechocystis_sp.                KIKAEGLIKDLKEAVAQ----E--DDAKIQTVMPELQQVLYSIGSNMYQQAGAEAGVGAP-------------GAGP---------EAG----TSSGGGD
Thermodesulfovibrio_yellowstonii RKRIEEAIEKCRRIK-D--TSN--DVNEIKAAVEELAKASHRVAEELYKKAGASQQGAG--------------------------------STTQSKKEE
Thermotoga_maritima              KSRLEDMIRELRDAINR----N--DIPKVKMLFDDLQKESMKIGEYLYKSATGGETSNQ-----------------------------------------
Thermus_thermophilus             RARLEAAIGKAKELVERDA-----PDPELKAATEELL-----------KAVEEYEK--GA-------------------------------QAASGKGPD
Vibrio_cholerae                  KAKIETAINELETAKKG----E--DKAEIDAKVQALMAAAQKLMEIAQQQAQAQ---------------------------------G-ANAGQSSAKED
Xanthomonas_campestris           IGKVEAALADLETAMKG----D--DKAQIEARSKTLEEAGQSLYAAAAAAEQGGN----------A--------------------DAASGNAQASKAAD
Yersinia_pestis                  KTAIEEAMKGLEAALKG----E--DKAEIEAKTQALVQVSGKLLEMAQQQQAAAG--------------------------------G-DAGDTSAKKED
                                                            :      .
```

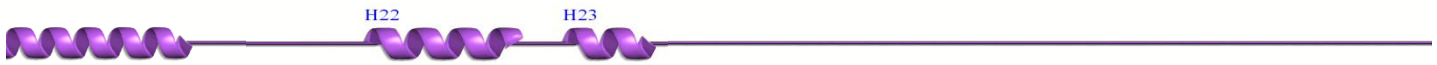

```
Acinetobacter_baumannii          GV--VDAEFTEVKDDKK---
Agrobacterium_tumefaciens        DV--VDADYEEIKDDKKSA-
Aquifex_aeolicus                 EGGEGEVEAKPVD-------
Bacillus_anthracis               NV--VDAEFEEVKEDK----
Bacillus_cereus                  NV--VDAEFEEVKEDK----
Bacillus_subtilis                NV--VDAEYEEVNDDQNKK-
Bacteroides_thetaiotaomicron     NV--QDADFEEVK-------
Bifidobacterium_longum           DT--VEAEVVDDDDDKDNK-
Bordetella_pertussis             NV--VDADFKEVKRDQ----
Borrelia_burgdorferi             EG--KEADYEVVDEDKK---
Bradyrhizobium_japonicum         DV--VDAEFTEVDDDKNNKK
Buchnera_aphidicola              NV--VDAEFEEIKDPKK---
Burkholderia_pseudomallei        DV--VDADFKEVKKD-----
Campylobacter_jejuni             DV--IDAEVE----------
Caulobacter_crescentus           GV--VDAEFEEVDDNKPAA-
Chlamydia_trachomatis            NI--EDADVEIVDKPE----
Chlorobium_tepidum               GA--VNAEYEVIDGDDK---
Clostridium_acetobutylicum       NV--VDADFKVDNDK-----
Clostridium_botulinum            NV--VDADFKVEDDK-----
Corynebacterium_glutamicum       NV--VDAEVVEDDAADNGED
Coxiella_burnetii                DV--VDAEFEEVKDEKKKDE
Deinococcus_radiodurans          DV--IDADFKPAE-------
Desulfovibrio_vulgaris           DV--VDADYTEVKK------
Dictyoglomus_turgidum            KT--IDGDYKVN--------
Enterococcus_faecalis            DV--VDADFEEVNGDDK---
Escherichia_coli                 DV--VDAEFEEVKDKK----
Flavobacterium_psychrophilum     DV--QDVEFEEVK-------
Francisella_tularensis           DV--VDADFEDVEDDKK---
Fusobacterium_nucleatum          EV--VD--------------
Geobacter_sulfurreducens         KV--VDADFEEVKDDKK---
Gloeobacter_violaceus            DV--IDAEFTETK-------
Haemophilus_influenzae           GV--VDAEFEEVKDNK----
Helicobacter_pylori              DV--IDAEVE----------
Koribacter_versatilis            GV--IDAEYVDVDEKK----
Lactobacillus_plantarum          NT--VDGDFEDLDKDKDKK-
Lactococcus_lactis               NT--FDGDFEESK-------
Leptospira_interrogans           KV--VDADYTVVDDEKK---
Listeria_monocytogenes           DV--VDAEFEEVNDDDKENK
Mesoplasma_florum                --------------------
Moorella_thermoacetica           NV--YDADYKVVDDDKKE--
Mycobacterium_smegmatis          NV--VDAEVVDDDAGKENK-
Mycobacterium_tuberculosis       DV--VDAEVVDDGREAK---
Mycoplasma_mycoides              --------------------
Mycoplasma_pneumoniae            --------------------
Neisseria_gonorrhoeae            DV--VDADFEEVKDDKK---
Neisseria_meningitidis           DV--VDADFEEVKDDKK---
Pseudomonas_aeruginosa           DV--VDAEFEEVKDNK----
Rhodobacter_sphaeroides          DI--VDADFEDLGENKRK--
Rhodopirellula_baltica           DA--IDAEFEVKE-------
Rhodospirillum_rubrum            GV--VDADFEEVDEKKGH--
Rickettsia_prowazekii            KV--VDADFQDVDKK-----
Salinibacter_ruber               DV--QDADYEVVDEGDDE--
Salmonella_typhimurium           DV--VDAEFEEVKDKK----
Shewanella_oneidensis            DV--VDAEFEEVKDDKK---
Shigella_dysenteriae             DV--VDAEFEEVKDKK----
Staphylococcus_aureus            TV--EDAEFKEVKDDDKK--
Streptococcus_pneumoniae         DV--VDGEFTEK--------
Streptomyces_coelicolor          DV--VDAEIVDDERKDGAA-
Synechocystis_sp.                DV--IDAEFSEPEK------
Thermodesulfovibrio_yellowstonii DV--IEAEVEDKDNK-----
Thermotoga_maritima              --------------------
Thermus_thermophilus             DV--IDADYKPAD-------
Vibrio_cholerae                  DV--VDAEFEEVNDDKK---
Xanthomonas_campestris           DV--VDAEFTEVKDDKKA--
Yersinia_pestis                  DV--VDAEFEEVKDKK----
```
