# Supplementary material for: DnaK as Antibiotic Target: Hot Spot Residues Analysis for Differential Inhibition of the Bacterial Protein in Comparison with the Human HSP70
Source: PLoS One. 2015 Apr 23;10(4):e0124563. doi: 10.1371/journal.pone.0124563 (PMC4408060; doi:10.1371/journal.pone.0124563)
Supplement: S2 Fig — (PDF) [file pone.0124563.s002.pdf]

```
HSP70-1A    ----------------MAKA--------------------------------AAIGIDLGTTYSCVGVFQHGKVEIIANDQGNRTTPSYVAFT-DTERLIGDAAKNQVA 61
HSP70-1L    -------------MATAKG---------------------------------IAIGIDLGTTYSCVGVFQHGKVEIIANDQGNRTTPSYVAFT-DTERLIGDAAKNQVA 63
HSP70-2     ---------------MSARG--------------------------------PAIGIDLGTTYSCVGVFQHGKVEIIANDQGNRTTPSYVAFT-DTERLIGDAAKNQVA 62
HSP70-4     ---------------------------------------------------MSVVGIDLGFQSCYVAVARAGGIETIANEYSDRCTPACISFG-PKNRSIGAAAKSQVI 58
HSP70-4L    ---------------------------------------------------MSVVGIDLGFLNCYIAVARSGGIETIANEYSDRCTPACISLG-SRTRAIGNAAKSQIV 58
HSP70-5     -MKLSLVAAMLLLSAARAEEEDKKEDVG------------------------TVVGIDLGTTYSCVGVFKNGRVEIIANDQGNRITPSYVAFTPEGERLIGDAAKNQLT 85
HSP70-6     -------------MQAPRE---------------------------------LAVGIDLGTTYSCVGVFQQGRVEILANDQGNRTTPSYVAFT-DTERLVGDAAKSQAA 63
HSP70-7     -------------MQAPRE---------------------------------LAVGIDLGTTYSCVGVFQQGRVEILANDQGNRTTPSYVAFT-DTERLVGDAAKSQAA 63
HSP70-8     ----------------MSKG--------------------------------PAVGIDLGTTYSCVGVFQHGKVEIIANDQGNRTTPSYVAFT-DTERLIGDAAKNQVA 61
HSP70-9     MISASRAAARLVGAAASRGPTAARHQDSWNGLSHEAFRLVSRRDYASEAIKGAVVGIDLGTTNSCVAVMEGKQAKVLENAEGARTTPSVVAFTADGERLVGMPAKRQAV 110
HSP70-12A   -----------------------------------------------------MADKEAGGS----DGPRET--APTSAYSSPARSLGDTGITPLSPSHIVNDTDSNVS 56
HSP70-12B   ----------------------------------------------------MLAVPEMGLQGLYIGSSPER--SPVPSPPGSPRTQESCGIAPLTPSQSP-KPEVRAP 57
HSP70-14    ---------------------------------------------------MAAIGVHLGCTSACVAVYKDGRAGVVANDAGDRVTPAVVAYS-ENEEIVGLAAKQSRI 58
                                                                      .*                     .   :    .            ..

HSP70-1A    LNPQNTVFDAKRLIGRKFGDPVVQSDMKHWPFQVINDGDKPKVQVSYKG-ETKAFYPEEISSMVLTKMKEIAEAYLGYPVTNAVITVPAYFNDSQRQATKDAGVIAGLN 169
HSP70-1L    MNPQNTVFDAKRLIGRKFNDPVVQADMKLWPFQVINEGGKPKVLVSYKG-ENKAFYPEEISSMVLTKLKETAEAFLGHPVTNAVITVPAYFNDSQRQATKDAGVIAGLN 171
HSP70-2     MNPTNTIFDAKRLIGRKFEDATVQSDMKHWPFRVVSEGGKPKVQVEYKG-ETKTFFPEEISSMVLTKMKEIAEAYLGGKVHSAVITVPAYFNDSQRQATKDAGTITGLN 170
HSP70-4     SNAKNTVQGFKRFHGRAFSDPFVEAEKSNLAYDIVQLPTGLTGIKVTYMEEERNFTTEQVTAMLLSKLKETAESVLKKPVVDCVVSVPCFYTDAERRSVMDATQIAGLN 167
HSP70-4L    TNVRNTIHGFKKLHGRSFDDPIVQTERIRLPYELQKMPNGSAGVKVRYLEEERPFAIEQVTGMLLAKLKETSENALKKPVADCVISIPSFFTDAERRSVMAAAQVAGLN 167
HSP70-5     SNPENTVFDAKRLIGRTWNDPSVQQDIKFLPFKVVEKKTKPYIQVDIGGGQTKTFAPEEISAMVLTKMKETAEAYLGKKVTHAVVTVPAYFNDAQRQATKDAGTIAGLN 195
HSP70-6     LNPHNTVFDAKRLIGRKFADTTVQSDMKHWPFRVVSEGGKPKVRVCYRG-EDKTFYPEEISSMVLSKMKETAEAYLGQPVKHAVITVPAYFNDSQRQATKDAGAIAGLN 171
HSP70-7     LNPHNTVFDAKRLIGRKFADTTVQSDMKHWPFQVVSEGGKPKVRVCYRG-EDKTFYPEEISSMVLSKMKETAEAYLGQPVKHAVITVPTYFSNSQRQATKDAGAIAGLK 171
HSP70-8     MNPTNTVFDAKRLIGRRFDDAVVQSDMKHWPFMVVNDAGRPKVQVEYKG-ETKSFYPEEVSSMVLTKMKEIAEAYLGKTVTNAVVTVPAYFNDSQRQATKDAGTIAGLN 149
HSP70-9     TNPNNTFYATKRLIGRRYDDPEVQKDIKNVPFKIVR-ASNGDAWVEAHG---KLYSPSQIGAFVLMKMKETAENYLGHTAKNAVITVPAYFNDSQRQATKDAGQISGLN 216
HSP70-12A   EQQSFLVVVAVDFGTTSSGYAYSFTKEPECIHVMRRWEGGDPGVSNQKTPTTILLTPERKFHSFGYAARDFYHDLDPNEAKQWLYLEKFKMK---LHTTGDLTMDTDLT 157
HSP70-12B   QQASFSVVVAIDFGTTSSGYAFSFASDPEAIHMMRKWEGGDPGVAHQKTPTCLLLTPEGAFHSFGYTARDYYHDLDPEEARDWLYFEKFKMK---IHSATDLTLKTQLE 161
HSP70-14    RNISNTVMKVKQILGRSSSDPQAQKYIAESKCLVIEKNGKLRYEIDTGE-ETKFVNPEDVARLIFSKMKETAHSVLGSDANDVVITVPFDFGEKQKNALGEAARAAGFN 166
             :    .     :       .            :                       .     .    ::  .      .   :            .:       : :

HSP70-1A    VLRIINEPTAAAIAYGLDRTG-----KGERNVLIFDLGGGTFDVSILTIDDGIFEVKATAGDTHLGGEDFDNRLVN-HFVEEFKRKHKKDISQNKRAVRRLRTACERAK 273
HSP70-1L    VLRIINEPTAAAIAYGLDKGG-----QGERHVLIFDLGGGTFDVSILTIDDGIFEVKATAGDTHLGGEDFDNRLVS-HFVEEFKRKHKKDISQNKRAVRRLRTACERAK 275
HSP70-2     VLRIINEPTAAAIAYGLDKKGC---AGGEKNVLIFDLGGGTFDVSILTIEDGIFEVKSTAGDTHLGGEDFDNRMVS-HLAEEFKRKHKKDIGPNKRAVRRLRTACERAK 276
HSP70-4     CLRLMNETTAVALAYGIYKQDLPALEEKPRNVVFVDMGHSAYQVSVCAFNRGKLKVLATAFDTTLGGRKFDEVLVN-HFCEEFGKKYKLDIKSKIRALLRLSQECEKLK 276
HSP70-4L    CLRLMNETTAVALAYGIYKQDLPPLDEKPRNVVFIDMGHSAYQVLVCAFNKGKLKVLATTFDPYLGGRNFDEALVD-YFCDEFKTKYKINVKENSRALLRLYQECEKLK 276
HSP70-5     VMRIINEPTAAAIAYGLDKRE------GEKNILVFDLGGGTFDVSLLTIDNGVFEVVATNGDTHLGGEDFDQRVME-HFIKLYKKKTGKDVRKDNRAVQKLRREVEKAK 298
HSP70-6     VLRIINEPTAAAIAYGLDRRG-----AGERNVLIFDLGGGTFDVSVLSIDAGVFEVKATAGDTHLGGEDFDNRLVN-HFMEEFRRKHGKDLSGNKRALRRLRTACERAK 275
HSP70-7     VLPIINEATAAAIAYGLDRRG-----AGKRNVLIFDLGGGTFDVSVLSIDAGVFEVKATAGDTHLGGEDFDNRLVN-HFMEEFRRKHGKDLSGNKRALRRLRTACERAK 275
HSP70-8     VLRIINEPTAAAIAYGLDKK-----VGAERNVLIFDLGGGTFDVSILTIEDGIFEVKSTAGDTHLGGEDFDNRMVN-HFIAEFKRKHKKDISENKRAVRRLRTACERAK 273
HSP70-9     VLRVINEPTAAALAYGLDKSE-------DKVIAVYDLGGGTFDISILEIQKGVFEVKSTNGDTFLGGEDFDQALLR-HIVKEFKRETGVDLTKDNMALQRVREAAEKAK 318
HSP70-12A   AANGKKVKALEIFAYALQYFK------EQALKELSDQAGSEFENSDVRWVITVPAIWKQPAKQFMRQAAYQAGLASPENSEQLIIALEPEAASIYCRKLRLHQMIELSS 261
HSP70-12B   AVNGKTMPALEVFAHALRFFR------EHALQELREQSPSLPEKDTVRWVLTVPAIWKQPAKQFMREAAYLAGLVSRENAEQLLIALEPEAASVYCRKLRLHQLLDLSG 265
HSP70-14    VLRLIHEPSAALLAYGIGQDS----PTGKSNILVFKLGGTSLSLSVMEVNSGIYRVLSTNTDDNIGGAHFTETLAQ-YLASEFQRSFKHDVRGNARAMMKLTNSAEVAK 271
                    :   :*:.:                 . . .    .            :     .  :    :   :               :         ::    :
HSP70-1A    RTLSS-STQASLEIDSLFEGID----FYTSITRARFEELCSDLFRSTLEPVEKALRDAKLDKAQIHDLVLVGGSTRIPKVQKLLQDFFNGRDLNKSINPDEAVAYGAAV 378
HSP70-1L    RTLSS-STQANLEIDSLYEGID----FYTSITRARFEELCADLFRGTLEPVEKALRDAKMDKAKIHDIVLVGGSTRIPKVQRLLQDYFNGRDLNKSINPDEAVAYGAAV 380
HSP70-2     RTLSS-STQASIEIDSLYEGVD----FYTSITRARFEELNADLFRGTLEPVEKALRDAKLDKGQIQEIVLVGGSTRIPKIQKLLQDFFNGKELNKSINPDEAVAYGAAV 381
HSP70-4     KLMSANASDLPLSIECFMNDVD----VSGTMNRGKFLEMCNDLLARVEPPLRSVLEQTKLKKEDIYAVEIVGGATRIPAVKEKISKFFG-KELSTTLNADEAVTRGCAL 381
HSP70-4L    KLMSANASDLPLNIECFMNDLD----VSSKMNRAQFEQLCASLLARVEPPLKAVMEQANLQREDISSIEIVGGATRIPAVKEQITKFFL-KDISTTLNADEAVARGCAL 381
HSP70-5     RALSS-QHQARIEIESFYEGED----FSETLTRAKFEELNMDLFRSTMKPVQKVLEDSDLKKSDIDEIVLVGGSTRIPKIQQLVKEFFNGKEPSRGINPDEAVAYGAAV 403
HSP70-6     RTLSS-STQATLEIDSLFEGVD----FYTSITRARFEELCSDLFRSTLEPVEKALRDAKLDKAQIHDVVLVGGSTRIPKVQKLLQDFFNGKELNKSINPDEAVAYGAAV 380
HSP70-7     RTPSS-STQATLEIDSLFEGVD----FYKSITRARFEELCSDLFRSTLEPVEKALRDAKLDKAQIHDFVLGGGLHSHP--------------------------QGAEV 351
HSP70-8     RTLSS-STQASIEIDSLYEGID----FYTSITRARFEELNADLFRGTLDPVEKALRDAKLDKSQIHDIVLVGGSTRIPKIQKLLQDFFNGKELNKSINPDEAVAYGAAV 378
HSP70-9     CELSS-SVQTDINLPYLTMDSSGPKHLNMKLTRAQFEGIVTDLIRRTIAPCQKAMQDAEVSKSDIGEVILVGGMTRMPKVQQTVQDLFG-RAPSKAVNPDEAVAIGAAI 426
HSP70-12A   KAAVNGYSGSD-TVGAGFTQAKEHIRRNRQSRTFLVENVIGEIWSELEEGDKYVVVDSGGGTVDLTVHQIRLPEGHLKELYKATGGPYGSLGVDYEFEKLLYKIFGEDF 370
HSP70-12B   RAPGGGRLGERRSIDSSFRQAREQLRRSRHSRTFLVESGVGELWAEMQAGDRYVVADCGGGTVDLTVHQLEQPHGTLKELYKASGGPYGAVGVDLAFEQLLCRIFGEDF 375
HSP70-14    HSLST-LGSANCFLDSLYEGQD----FDCNVSRARFELLCSPLFNKCIEAIRGLLDQNGFTADDINKVVLCGGSSRIPKLQQLIKDLFPAVELLNSIPPDEVIPIGAAI 376
                          :                    .      :        .  : :      .:    :                                   *  .

HSP70-1A    QAAILMGDKSENVQDLLLLDVAP---LSLGLETAGG-VMTALIKRNSTIPTKQTQIFTTYSDNQPGVLIQVYEG--ERAMTKDNNLLGRFELSGIPPAPRG-VPQIEVT 477
HSP70-1L    QAAILMGDKSEKVQDLLLLDVAP---LSLGLETAGG-VMTALIKRNSTIPTKQTQIFTTYSDNQPGVLIQVYEG--ERAMTKDNNLLGRFDLTGIPPAPRG-VPQIEVT 479
HSP70-2     QAAILIGDKSENVQDLLLLDVTP---LSLGIETAGG-VMTPLIKRNTTIPTKQTQTFTTYSDNQSSVLVQVYEG--ERAMTKDNNLLGKFDLTGIPPAPRG-VPQIEVT 480
HSP70-4     QCAILSPAFKVREFSITDVVPYP-ISLRWNSPAEEGSSDCEVFSKNHAAPFSKVLTFYRK---EPFTLEAYYSS--PQDLPYPDPAIAQFSVQKVTPQSDGSSSKVKVK 481
HSP70-4L    QCAILSPAFKVREFSITDLVPYS-ITLRWKTSFEDGSGECEVFCKNHPAPFSKVITFHKK---EPFELEAFYTN--LHEVPYPDARIGSFTIQNVFPQSDGDSSKVKVK 481
HSP70-5     QAGVLSGD--QDTGDLVLLDVCP---LTLGIETVGG-VMTKLIPRNTVVPTKKSQIFSTASDNQPTVTIKVYEG--ERPLTKDNHLLGTFDLTGIPPAPRG-VPQIEVT 500
HSP70-6     QAAVLMGDKCEKVQDLLLLDVAP---LSLGLETAGG-VMTTLIQRNATIPTKQTQTFTTYSDNQPGVFIQVYEG--ERAMTKDNNLLGRFELSGIPPAPRG-VPQIEVT 479
HSP70-7     AAGLLQRQGAEQEHQP---------------------------------------------------------------------------------------------  367
HSP70-8     QAAILSGDKSENVQDLLLLDVTP---LSLGIETAGG-VMTVLIKRNTTIPTKQTQTFTTYSDNQPGVLIQVYEG--ERAMTKDNNLLGKFELTGIPPAPRG-VPQIEVT 477
HSP70-9     QGGVLAGD----VTDVLLLDVTP---LSLGIETLGG-VFTKLINRNTTIPTKKSQVFSTAADGQTQVEIKVCQG--EREMAGDNKLLGQFTLIGIPPAPRG-VPQIEVT 521
HSP70-12A   IEQFKIKRPAAWVDLMIAFESRK---RAAAPDRTNPLNITLPFSFIDYYKKFRGHSVEHALRKSNVDFVKWSSQGMLRMSPDAMNALFKPTIDSIIEHLRDLFQKPEVS 473
HSP70-12B   IATFKRQRPAAWVDLTIAFEARK---RTAGPHRAGALNISLPFSFIDFYRKQRGHNVETALRRSSVNFVKWSSQGMLRMSCEAMNELFQPTVSGIIQHIEALLARPEVQ 478
HSP70-14    EAGILIGKENLLVEDSLMIECSARDILVKGVDESGASRFTVLFPSGTPLPARRQHTLQAPGSISSVCLELYESD--GKNSAKEETKFAQVVLQDLDKKENG-LRDILAV 479
               .

HSP70-1A    FDIDANGILNVTAT------------------------------------DKSTGKANKITITNDKGRLSKEE------------------------------------ 514
HSP70-1L    FDIDANGILNVTAT------------------------------------DKSTGKVNKITITNDKGRLSKEE------------------------------------ 516
HSP70-2     FDIDANGILNVTAA------------------------------------DKSTGKENKITITNDKGRLSKDD------------------------------------ 517
HSP70-4     VRVNVHGIFSVSSASLVEVHKSE--ENEEPMETDQNAKEE-----EKMQVDQEEPHVEEQQQQTPAENKAESEEMETSQAGSKDKKMDQPPQAKKAKVKTSTVDLPIEN 583
HSP70-4L    VRVNIHGIFSVASASVIEKQNLEGDHSDAPMETETSFKNENKDNMDKMQVDQEEGHQKCHAEHTPEEEIDHTGAKTKSAVSDKQDRLNQT--LKKGKVKS--IDLPIQS 586
HSP70-5     FEIDVNGILRVTAE------------------------------------DKGTGNKNKITITNDQNRLTPEE------------------------------------ 537
HSP70-6     FDIDANGILSVTAT------------------------------------DRSTGKANKITITNDKGRLSKEE------------------------------------ 516
HSP70-7     -------------------------------------------------------------------------------------------------------------
HSP70-8     FDIDANGILNVSAV------------------------------------DKSTGKENKITITNDKGRLSKED------------------------------------ 514
HSP70-9     FDIDANGIVHVSAK------------------------------------DKGTGREQQIVIQSS-GGLSKDD------------------------------------ 557
HSP70-12A   TVKFLFLVGGFAEAP-----------------------------LLQQAVQAAFGDQ-CRIIIPQDVGLTILKG----------------------------------- 517
HSP70-12B   GVKLLFLVGGFAESA-----------------------------VLQHAVQAALGARGLRVVVPHDVGLTILKG----------------------------------- 523
HSP70-14    LTMKRDGSLHVTCT------------------------------------DQETGKCEAISIEIAS------------------------------------------- 509


HSP70-1A    ----------IERMVQEAEKYKAEDEVQRERVSAKNALESYAFNMKSAVED---------------------------------------------------------- 555
HSP70-1L    ----------IERMVLDAEKYKAEDEVQREKIAAKNALESYAFNMKSVVSD---------------------------------------------------------- 557
HSP70-2     ----------IDRMVQEAERYKSEDEANRDRVAAKNALESYTYNIKQTVED---------------------------------------------------------- 558
HSP70-4     QLLWQIDREMLNLYIENEGKMIMQDKLEKERNDAKNAVEEYVYEMRDKLSGEYEKFVSEDDRNSFTLKLEDTENWLYEDGEDQPKQVYVDKLAELKNLGQPIKIRFQES 692
HSP70-4L    SLCRQLGQDLLNSYIENEGKMIMQDKLEKERNDAKNAVEEYVYDFRDRLGTVYEKFITPEDLSKLSAVLEDTENWLYEDGEDQPKQVYVDKLQELKKYGQPIQMKYMEH 695
HSP70-5     ----------IERMVNDAEKFAEEDKKLKERIDTRNELESYAYSLKNQIGDK--------------------------------------------------------- 579
HSP70-6     ----------VERMVHEAEQYKAEDEAQRDRVAAKNSLEAHVFHVKGSLQE---------------------------------------------------------- 557
HSP70-7     -------------------------------------------------------------------------------------------------------------
HSP70-8     ----------IERMVQEAEKYKAEDEKQRDKVSSKNSLESYAFNMKATVED---------------------------------------------------------- 555
HSP70-9     ----------IENMVKNAEKYAEEDRRKKERVEAVNMAEGIIHDTETKMEE---------------------------------------------------------- 598
HSP70-12A   ----------AVLFGLDPAVIKVRRSPLTYGVGVLNRYVEGKHPPEKLLVKDGTR------------------------------------------------------ 562
HSP70-12B   ----------AVLFGQAPGVVRVRRSPLTYGVGVLNRFVPGRHPPEKLLVRDGRR------------------------------------------------------ 568
HSP70-14    -------------------------------------------------------------------------------------------------------------


HSP70-1A    ---------------------------EGLKGKISEADKKKVLDKCQEVISWLDANTLAEKD------------EFEHKRKELEQVCNPIISGLYQGA-----GGPGPG 620
HSP70-1L    ---------------------------EGLKGKISESDKNKILDKCNELLSWLEVNQLAEKD------------EFDHKRKELEQMCNPIITKLYQG------GCTGP- 620
HSP70-2     ---------------------------EKLRGKISEQDKNKILDKCQEVINWLDRNQMAEKD------------EYEHKQKELERVCNPIISKLYQ---GGPGGGSG-- 623
HSP70-4     EERPKLFEELGKQIQQYMKIISSFKNKEDQYDHLDAADMTKVEKSTNEAMEWMNNKLNLQNKQSLTMDPVVKSKEIEAKIKELTSTCSPIISKPKPKVEPPKEE-QKNA 800
HSP70-4L    EERPKALNDLGKKIQLVMKVIEAYRNKDERYDHLDPTEMEKVEKCISDAMSWLNSKMNAQNKLSLTQDPVVKVSEIVAKSKELDNFCNPIIYKPKPKAEVPEDKPKANS 804
HSP70-5     ---------------------------EKLGGKLSSEDKETMEKAVEEKIEWLESHQDADIE------------DFKAKKKELEEIVQPIISKLYG------------- 636
HSP70-6     ---------------------------ESLRDKIPEEDRRKMQDKCREVLAWLEHNQLAEKE------------EYEHQKRELEQICRPIFSRLYGGP-----GVPGGS 622
HSP70-7     -------------------------------------------------------------------------------------------------------------
HSP70-8     ---------------------------EKLQGKINDEDKQKILDKCNEIINWLDKNQTAEKE------------EFEHQQKELEKVCNPIITKLYQSAGGMPGGMPGGF 625
HSP70-9     -----------------------------FKDQLPADECNKLKEEISKMRELLARKDSETGE------------NIRQAASSLQQASLKLFEMAYKK--------MASE 658
HSP70-12A   ------------------------WCTDVFDKFISADQSVALGELVKRSYTPAKPSQLVIVIN-----------IYSSEHDNVSFITDPGVKKCGTLRLDLT-----GT 632
HSP70-12B   ------------------------WCTDVFERFVAAEQSVALGEEVRRSYCPARPGQRRVLIN-----------LYCCAAEDARFITDPGVRKCGALSLELEPADCGQD 642
HSP70-14    -------------------------------------------------------------------------------------------------------------


HSP70-1A    GFGAQGPKGGSGSGPTIEEVD----------------------- 641
HSP70-1L    ACGTGYVPGRPATGPTIEEVD----------------------- 641
HSP70-2     -GGGS----GASGGPTIEEVD----------------------- 639
HSP70-4     EQNGPVDGQGDNPGPQAAEQGTDTAVPSDSDKKLPEMDID---- 840
HSP70-4L    EHNGPMDGQSG-----TETKSDSTKDSSQHTKSSGEMEVD---- 839
HSP70-5     --SAGPPPTGEEDTAEKDEL------------------------ 654
HSP70-6     SCGTQARQGDPSTGPIIEEVD----------------------- 643
HSP70-7     --------------------------------------------
HSP70-8     PGGGAPPSGGASSGPTIEEVD----------------------- 646
HSP70-9     REGSGSSGTGEQKEDQKEEKQ----------------------- 679
HSP70-12A   SGTAVPARREIQTLMQFGDTEIKATAIDIATSKSVKVGIDFLNY 675
HSP70-12B   TAGAPPGRREIRAAMQFGDTEIKVTAVDVSTNRSVRASIDFLSN 686
HSP70-14    --------------------------------------------
```
